# A tunable multi-timescale Indium-Gallium-Zinc-Oxide thin-film transistor neuron towards hybrid solutions for spiking neuromorphic applications

**DOI:** 10.1038/s44172-024-00248-7

**Published:** 2024-07-23

**Authors:** Mauricio Velazquez Lopez, Bernabe Linares-Barranco, Jua Lee, Hamidreza Erfanijazi, Alberto Patino-Saucedo, Manolis Sifalakis, Francky Catthoor, Kris Myny

**Affiliations:** 1 EPIC, Large Area Thin-film Transistor Electronics, imec, Kapeldreef 75, 3001 Leuven, Belgium; 2 ES&S, COSIC, ESAT, KU Leuven, 3590 Diepenbeek, Belgium; 3 Instituto de Microelectrónica de Sevilla, IMSE-CNM, (CSIC Universidad de Sevilla), 41092 Sevilla, Spain; 4 School of Information and Communication Engineering, Sungkyunkwan University (SKKU), 16419 Seoul, South Korea; 5 Imec The Netherlands, High-Tech Campus 31, 5656 Eindhoven, The Netherlands; 6 ESAT, KU Leuven, 3000 Leuven, Belgium

**Keywords:** Electrical and electronic engineering, Electronics, photonics and device physics, Nanoscience and technology

## Abstract

Spiking neural network algorithms require fine-tuned neuromorphic hardware to increase their effectiveness. Such hardware, mainly digital, is typically built on mature silicon nodes. Future artificial intelligence applications will demand the execution of tasks with increasing complexity and over timescales spanning several decades. The multi-timescale requirements for certain tasks cannot be attained effectively enough through the existing silicon-based solutions. Indium-Gallium-Zinc-Oxide thin-film transistors can alleviate the timescale-related shortcomings of silicon platforms thanks to their bellow atto-ampere leakage currents. These small currents enable wide timescale ranges, far beyond what has been feasible through various emerging technologies. Here we have estimated and exploited these low leakage currents to create a multi-timescale neuron that integrates information spanning a range of 7 orders of magnitude and assessed its advantages in larger networks. The multi-timescale ability of this neuron can be utilized together with silicon to create hybrid spiking neural networks capable of effectively executing more complex tasks than their single-technology counterparts.

## Abstract (web summary)

Mauricio Velazquez Lopez and colleagues fabricate a neuromorphic node with a response time that spans a range of 7 orders of magnitude. Their technology is compatible with complementary metal-oxide semiconductors, which makes it suitable for a variety of machine learning tasks.

## Introduction

Thin-film transistors (TFTs) are currently the dominant technology in the display industry thanks to the possibility of manufacturing them at low costs over large areas on top of glass, flexible and stretchable substrates^1^. The Internet-of-Things (IoT) brought to the forefront the potential of TFTs to generate valuable technological developments for applications other than the display industry^2^. Examples include flexible micropocessors^3,4^, capacitive touchscreen tags^5^, power management blocks for wearable large area sensor arrays^6^, ultrasonic phased arrays for medical imaging^7^, and bioelectronic patches for mapping of electrophysiological activities^8^. Artificial intelligence (AI) and neuromorphic circuits presents another area where TFTs have recently started to demonstrate their potential. Applications such as an artificial afferent nerve designed on flexible organic electronics^9^, a synapse network utilizing polymer-based memristive devices^10^ and a hardwired machine learning processing engine applied to sweat odour classification fabricated with IGZO TFTs^11^ are shown in the literature.

Streaming edge-AI applications like bio-signal processing for point-of-care (PoC) devices require specialized neuromorphic hardware. Traditional convolutional neural network (CNN) and deep neural network (DNN) algorithms are not that well suited for these target domain requirements since for such highly complex applications, they can only handle the streaming sample sequence with quite large hardware overheads. Those hardware overheads are not compatible with ultra-low power edge platform specifications. Therefore, another part of the research community has studied other brain-inspired alternatives, especially different forms of spiking neural networks (SNNs). In today’s literature, these platforms have been largely digital in nature^12–16^, realized on very mature CMOS nodes. Still, non-digital platforms have also been widely studied in the last decade^16–20^. In spite of these recent developments, AI applications continue to demand the execution of progressively complex tasks throughout diverse and extended timescales with a wide range (up to many decades) which the existing CMOS-based spiking neuromorphic platforms alone cannot tackle in a sufficiently effective way.

We can illustrate this by the following case study. SNNs are specifically well fit for the processing of temporary encoded data such as bio signals^16^. The most vital bio signals utilized to demonstrate these networks are electrocardiography (ECG) and electromyography (EMG)^21^. Recently, electrocorticography (ECoG) real-time recordings have also been processed by an SNN^22^. Despite the maturity of CMOS technology and the successful development of advanced SNNs capable of performing these intricate jobs, the implementation of these networks is already challenging when the time constants of the CMOS chip are by definition faster than the bio signals to be monitored^23,24^. A well-known biological application such as speech recognition can quickly become very challenging from the moment the system must perform not only mere speech recognition but semantic understanding of speech where short voice samples (milliseconds), words/sentences (minutes) as well as interpretation of long texts or discussion topics (hours) must be handled simultaneously for semantic contextualization; the two latter timescales can be very expensive to implement in CMOS hardware where long time constants can cost valuable silicon area if generated through passive circuits such as RC-filters^23^, or alternatively with the use of huge memory banks to support the many decades. Similarly, the emerging non-volatile memory (NVM) solutions can more natively support the analogue timescale concept, but it has been shown to be extremely difficult to go beyond 2 or 3 decades of time-scale range^25,26^ with one recent exception of 6 decades in simulation^27^. Further complexity is conceivable when considering disease diagnosis where sensing must be performed by sensors in milliseconds, drug effects must be monitored in seconds to minutes (or hours) and treatments are evaluated in days to months (or even years).

**Fig. 1:**
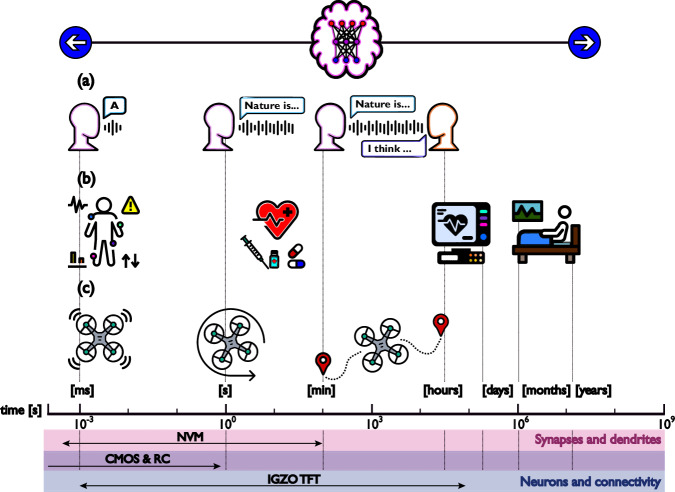
Applications requiring AI systems with several timescales and the timescales made available by NVM devices, silicon CMOS including its RC-constants, and IGZO TFTs. **a** Long-term speech recognition. **b** Disease diagnosis. **c** Autonomous robots. AI Artificial Intelligence, NVM Non-Volatile Memory, CMOS Complementary Metal-Oxide Semiconductor, RC Resistor Capacitor, IGZO TFT Indium-Gallium-Zinc-Oxide Thin-Film Transistor.

The same observations can be extracted from streaming applications not related to healthcare. Autonomous vehicles for example, must continuously monitor their environment in millisecond sampling periods, react in fractions of seconds to minutes when steering and be capable of planning long trajectories (hours). A remarkably comprehensive analysis of timescales for computing systems including spiking neuromorphic hardware is presented by Jaeger et al.^23^. A summary of the applications mentioned above along with an approximation of the timescales made available by the different types of technologies we have mentioned^23–27^ are illustrated in Fig. 1. Additionally, the parts of spiking neuromorphic hardware for which said technologies are typically used is also shown, each technology has its own strengths and may cover different timescale ranges, Fig. 1 only illustrates their most common role and the timescales they can enclose. Results for IGZO TFTs are added based on the results presented in this paper.

Researchers have tried to diversify the fast time constants of silicon neuromorphic hardware through various methods. These techniques depend on the type of hardware available, either analogue neuromorphic or digitally simulated platforms. Digitally simulated CNN and DNN platforms provide flexibility (at the trade-off of computational resources) for the implementation of timescale variations. This flexibility involves the online adaptation of the time constants in the neurons conforming the simulated network, which is in fact a possible solution of the time warping problem whose explanation falls out of the scope of this paper. However, an extensive description of the problem and its possible solutions are addressed by Lukoševičius et al.^24^. Modification of the timescales of neurons conforming an analogue SNN is less straightforward. Fast time constants are the strong suit of CMOS technology, slow time constants on the other hand are harder to generate. Common solutions include the exploitation of nano-ampere to pico-ampere currents by means of transistors biased in their sub-threshold region^28^. These currents can be lowered to femto-ampere levels through source voltage shifting of these transistors^29^. Algorithms to slow down analogue SNNs have also been explored albeit not as extensively as the ones applicable to their digital counterparts. One proven solution is Reservoir Transfer which enables an SNN to integrate information over a time period longer than the timescales of its individual neurons^30^. In summary, one can change the timescales close to the neurons themselves or utilize an algorithm that allows integration of information over longer time spans. Literature suggests that the introduction of several timescales within the neurons of a network increases problem solving performance^31,32^ and in some cases it can result in less complex/more compact networks^33^.

Neurons in silicon CMOS technology have been trying to generate bio-compatible analogue timescales and have been successful only in the kHz to tens of Hz range^34–40^ where they are faced with either area constraints or limited by the high leakage of this technology. On the other hand, more complex CMOS circuits such as voltage-controlled oscillators (VCO) and neural oscillators have been able to generate a wide range of timescales of up to 9 orders of magnitude^41,42^. However, these circuits extend their oscillation range in the MHz domain away from bio-compatible timescales, similar to the NVM neurons presented by Goda et al.^27^. Thus, demonstrating the extreme low leakage currents of IGZO TFTs^43,44^ in the context of a neuromorphic circuit such as a neuron would bring an important opportunity forward enabling deep sub-Hz timescales in future CMOS, NVM and TFT hybrid spiking neuromorphic circuits.

Neuromorphic circuits, including neuron circuits themselves, can benefit not only from the exceedingly low leakage currents of IGZO TFTs but also from continuous technological developments suggesting narrow process variations^4^ and limited leakage current variations even at considerably high temperatures of up to 150 ˚C^44^. Process and temperature variations can affect other transistor traits such as the threshold voltage of IGZO TFTs which can be addressed via biasing voltages or in some cases via the addition of an additional back-gate (BG) to the transistors^44^. Recently, the spatiotemporal capabilities of a single synaptic TFT in a motion recognition nerve have been demonstrated. One of the suggested applications for this nerve was the recognition of drone flight modes which requires timescales in the order of seconds^45^. This suggestion aligns with the previously discussed complex applications if one would desire full autonomous operation of the drone. However, this example solely uses a single TFT as a synapse.

The number of neurons fully designed using only TFTs is still limited. Some of the reported thin-film neurons until now have already shown bio-compatible timescales on the order of milliseconds^46,47^ to seconds^47^. Our previous work, even though not optimized for leakage current exploitation, showed a timescale range of up to 3 orders of magnitude on a dual-gate (DG) IGZO TFT 5 µm node by varying a single BG bias voltage^47^.

In this paper, we exploit the extremely low levels of leakage of a single-gate, n-type only IGZO TFT 0.8 µm node from the foundry Pragmatic^4^ to design a neuron capable of integrating information over a range of timescales 7 orders of magnitude wide based on the excitation speed of its synapse and adjustable by means of a single bias voltage. It is important to note that the final targeted system is a hybrid combination of Si CMOS and IGZO, however, to illustrate the viability of our fundamental concept this research focused on an initial implementation using only IGZO TFTs. This timescale range is to the author’s best knowledge, one of the longest ones for a spiking neuron circuit and possibly the slowest (hundreds of µHz) firing rate reported to date. We also demonstrate the capabilities of this technology to generate an even broader range of timescales of up to 10 decades through the oscillations of a current-controlled oscillation (CCO) circuit. A full SNN simulation presents then the improved long-term memory and reduced network complexity resulting from utilizing multiple neurons with multi-timescale capabilities, giving a glance into the future of hybrid CMOS + TFT neurons. In addition, we have performed extensive measurements on fabricated CCOs and neuron prototypes that required us to solve additional challenges in the measurement procedure which is our additional contribution of this paper.

This paper is organized as follows: a first order approximation of Pragmatic 0.8 µm TFTs’ leakage current levels is made through direct measurements. Then, a second more accurate estimation is implemented indirectly through a series of two CCO circuits, where one of them is used as a building block of the final multi-timescale TFT neuron. Supplementary material is provided about the second CCO showing the possibility of a wider range of timescales through its oscillations and illustrating the future challenges of IGZO-based neuromorphic circuits. Finally, we present a full SNN simulation where all neurons are modelled following the behaviour of the multi-timescale TFT neuron and discuss how the wide range of timescales made available by it opens the possibility of hybrid SNNs where IGZO TFTs help cover timescales that would otherwise be very difficult to attain using only silicon neurons. Current challenges and limitations of the TFT neuron along with future plans to realize a CMOS + TFT hybrid neuron are also discussed. The authors believe that hybrid (CMOS, NVM and TFT) neuromorphic circuits are the key towards networks capable of executing extremely complex tasks over bio-compatible timescales.

## Results

### IGZO TFT leakage current approximation

The lowest leakage current reported for IGZO TFTs is 50 yA µm^−1^ (“y” being the prefix yokto and representing 10^−24^)^43^. This incredible property of IGZO was seldom exploited in circuits outside the display industry which benefits from the resulting high retention times^48^. Some applications outside the display industry that have been exploiting these ultra-low-level currents include “normally-off” computing chips^49^ and lately research in capacitorless DRAM^50^.

**Fig. 2:**
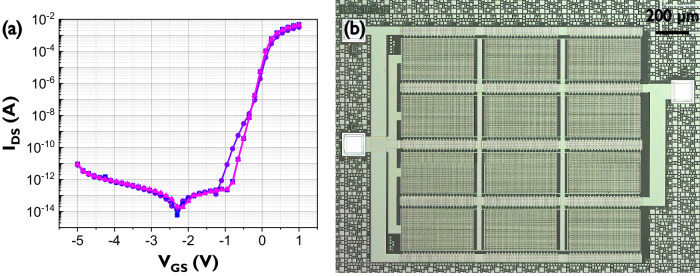
Transfer curve, source gate voltage (*V*_GS_) vs source drain current (*I*_DS_), of 12k minimum size n-type IGZO TFTs. **a** Superposed transfer characteristics for *V*_DS_ = 0.1 V. The “dip” in the off-region stems from gate leakage present due to the large size of the TFT. **b** Micrograph of the device formed by all 12k minimum size TFTs. IGZO TFT Indium-Gallium-Zinc-Oxide thin-film transistor.

A TFT neuron can potentially take advantage of these low currents and utilize them to generate a wide range of timescales. However, approximating such low current levels is very challenging and actual current levels will depend on the IGZO stack being measured. Furthermore, the goal is not only the measurement of leakage current but mainly its exploitation for a controllable neuron where we configure the current timescale to any value in the potentially supported range, which is far from obvious. In order to reach this goal, more sophisticated circuits which add complexity by including the interaction of extremely low currents with other circuit elements were explored. Figure 2a shows the superposed transfer characteristics of several devices each connecting >12,000 minimum sized TFTs (W/L = 5 µm /0.8 µm) in parallel. This transfer characteristic shows off-current levels close to the noise floor of the characterization tool. The off-current of all devices remains below 1 pA for all 12 thousand minimum size transistors, this in turn puts the magnitude of the off-current of a single TFT at aA levels (“a” being the prefix atto and representing 10^−18^).

**Fig. 3:**
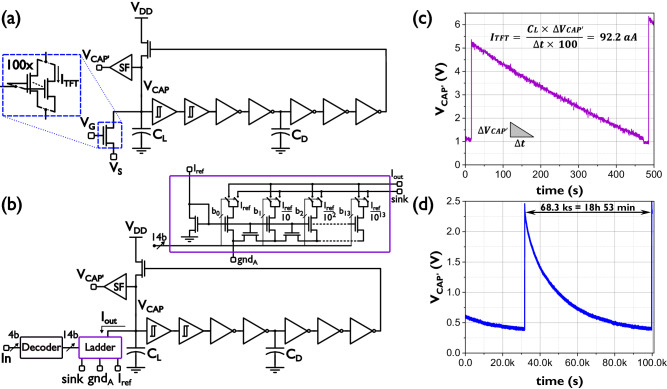
Current controlled oscillators (CCO) used for TFT leakage current approximations. *V*_CAP_ is the voltage of leakage capacitor *C*_L_, *C*_D_ is the fixed delay capacitor and *V*_CAP_, is the output of source follower (SF). **a** CCO controlled by the leakage current *I*_TFT_ of 100 TFTs in parallel (*V*_GS_ < −1 V). **b** CCO controlled by the current sunk via a 14-bit ladder circuit (bits b_0_ to b_13_) through the current of node *I*_out_. *I*_ref_ is the reference current of the ladder circuit, node “gnd_A_^”^ is a separate analogue ground and “sink” is a current sinking node. “In” is the 4-bit word utilized by the decoder to control the ladder circuit. **c** Longest capacitor discharge curve obtained by the CCO in (**a**) where ∆*V*_CAP’_ is the difference between the maximum and minimum values of *V*_CAP’_ and ∆*t* the time span in between said values. **d** Longest capacitor discharge curve obtained by the CCO in (**b**). TFT Thin-Film Transistor.

To put these leakage currents in context of a larger circuit and to approximate more accurately their value, two current-controlled oscillation (CCO) circuits shown in Fig. 3 were designed, fabricated and characterized.

Both CCOs are formed by a chain of resistive-load logic (R-logic) inverters (Supplementary Fig. 1) whose high gain and unbalanced transfer characteristic^3,51^ allow the circuits to oscillate even at the very low frequencies generated by discharging the leakage capacitor *C*_L_ with extremely low currents. Input R-Schmitt triggers with fixed hysteresis windows (Supplementary Fig. 2) also serve this purpose and together with capacitor *C*_D_ introduce fixed delays in the chain that stabilize the oscillations and maintain the width of the feedback pulse constant and wide enough to effectively recharge capacitor *C*_L_ and thus recommence oscillations. The difference between both circuits is the way in which the leakage current that discharges *C*_L_ is controlled. The CCO in Fig. 3a uses 100 TFTs (W/L = 20 µm/0.8 µm) in parallel as discharge elements while the CCO in Fig. 3b utilizes a ladder circuit controlled by a 4-bit decoder. The ladder circuit consists of several branches each one of them dividing the previous branch current by a factor N depending on the transistor sizing^29^.

In this case a 1 µA reference current (*I*_ref_) is divided by a factor N ~ 10 by each one of the 13 branches in the circuit. The ladder circuit can therefore generate currents in the order of a single minimum size TFT’s leakage current (~aA). Figure 3c, d present the capacitor discharge voltage corresponding to the slowest oscillation attained by each CCO. A linear discharge approximation applied to the voltage in Fig. 3c places the total discharge current at 92.2 fA and therefore the leakage current of a single 20 µm /0.8 µm TFT at 92.2 aA. On the other hand, an RC discharge curve fitting in Fig. 3d approximates a discharge current of 6 aA for the 9^th^ branch of the ladder circuit during the last hours of discharge. The aforementioned currents generated oscillations as long as 470 s (~8 min) and 68 ks (18 h 53 min) respectively. With the fastest oscillations measured at around 4 µs for both CCOs, a conservative oscillation timescale range of 7–8 orders of magnitude was demonstrated by the CCO in Fig. 3a while the CCO in Fig. 3b shows that a much wider range of over 10 decades is possible (see supplementary material). These results illustrate the incredible advantage that IGZO TFTs have at generating a very broad range of timescales that are bio-compatible with brain activity, short-term memory, and even long-term memory time spans, which in turn suggests that IGZO TFTs can help compensate for CMOS neurons’ limitations at longer timescales and pave the way for future hybrid solutions capable of solving increasingly complex tasks during long periods of time.

### Multi-timescale TFT neuron

**Fig. 4:**
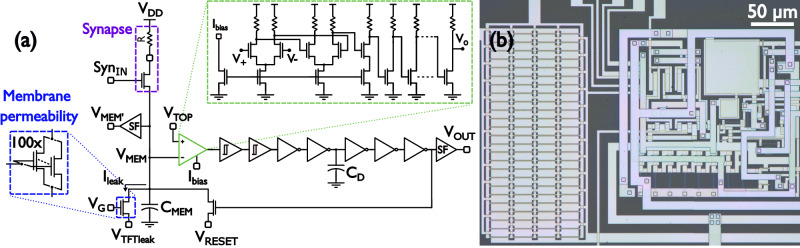
Multi-timescale TFT neuron. **a** Circuit schematic. **b** Micrograph, total area 416 µm by 254 µm from which an area of 254 µm by 213 µm ( ~ 48%) is occupied by the leak TFTs on the left side. Membrane capacitor *C*_MEM_ and fixed delay capacitor *C*_D_ are 1 pF and 10 pF respectively. The external synapse resistor (R) was fixed at 10 MΩ. *V*_TOP_, *I*_bias_, and *V*_o_ are the threshold voltage, bias current, and output node of the comparator while its positive and negative inputs are denoted by *V*_+_ and *V*_-_. *V*_MEM’_ is the output of the source follower (SF) following the membrane potential *V*_MEM_ and *V*_OUT_ is the output of the neuron buffered by an additional SF circuit. TFT Thin-Film Transistor.

The proposed multi-timescale TFT neuron circuit including all the elements explained before is shown in Fig. 4a. The core of the circuit is similar to the previously discussed CCOs with the addition of a comparator at the beginning of the chain. Capacitor *C*_L_ in Fig. 3 is now the membrane capacitor *C*_MEM_, and the output of the inverter chain is this time connected to a reset transistor across *C*_MEM_. Membrane voltage *V*_MEM_ will oscillate between *V*_TOP_ and *V*_RESET_. The synapse is formed by a simple 1T1R circuit with the resistor being external for flexibility. The membrane permeability is modelled by the leakage current of 100 TFTs (W/L = 20 µm/0.8 µm) in parallel and can be controlled by voltages *V*_G_ and *V*_TFTleak_. The synapse transistor is operated by applying short digital spikes at node “Syn_IN_”. It is important to guarantee that during inter-spike times, this synapse transistor does not introduce any leakage, by making sure its *V*_GS_ voltage is highly negative (*V*_GS_ < −1 V). Since the synapse transistor’s gate voltage is 0 V during inter-spike intervals, the minimum *V*_MEM_ voltage (set by *V*_RESET_) should be high enough to prevent this undesired leakage contribution.

A pulse train with constant frequency and pulse width (100 ns) present at the input of the synapse will deliver charge packages to *C*_MEM_ which will increase the membrane potential *V*_MEM_. The multi-timescale TFT neuron will spike once whenever *V*_MEM_ surpasses the comparator’s threshold voltage determined by *V*_TOP_. Both *V*_MEM_ and the neuron’s output are monitored by minimum size source follower circuits utilized as buffers. The neuron’s output spike will reset *V*_MEM_ to a voltage determined by *V*_RESET_ which will prepare the neuron to fire again.

The exceptionally low current with which *C*_MEM_ can be discharged, together with the frequency and size of the charge packets coming from the neuron’s synapse will generate spikes spaced by a very diverse and wide range of time between spikes (TBS). In other words, the multi-timescale TFT neuron will adapt the TBS of its output spikes according to the frequency of excitation present at the synapse for a fixed level of membrane permeability, synaptic charge packet, and voltage swing (Δ*V*_MEM_ = *V*_TOP_ - *V*_RESET_). Thus, the TBS can be described by the equation:

1 $${{\rm{TBS}}}=\frac{n{q}_{{{\rm{syn}}}}-{C}_{{{\rm{MEM}}}}{\Delta V}_{{{\rm{MEM}}}}}{{I}_{{{\rm{leak}}}}},$$

where *n* is number of synaptic spikes during one TBS, *q*_syn_ is the charge packet size (which depends on external resistor *R*, the synaptic pulse width, and the instantaneous value of *V*_MEM_), *C*_MEM_ is the membrane capacitor, Δ*V*_MEM_ is the voltage swing and *I*_leak_ is the leakage current of the 100 TFTs that model the membrane’s permeability. Furthermore, for a fixed excitation frequency at the synapse, changes in membrane permeability will result in yet additional timescales in between spikes. This acts as supplementary degree of freedom and effectively increases the number of possible timescales that the multi-timescale TFT neuron can achieve.

**Fig. 5:**
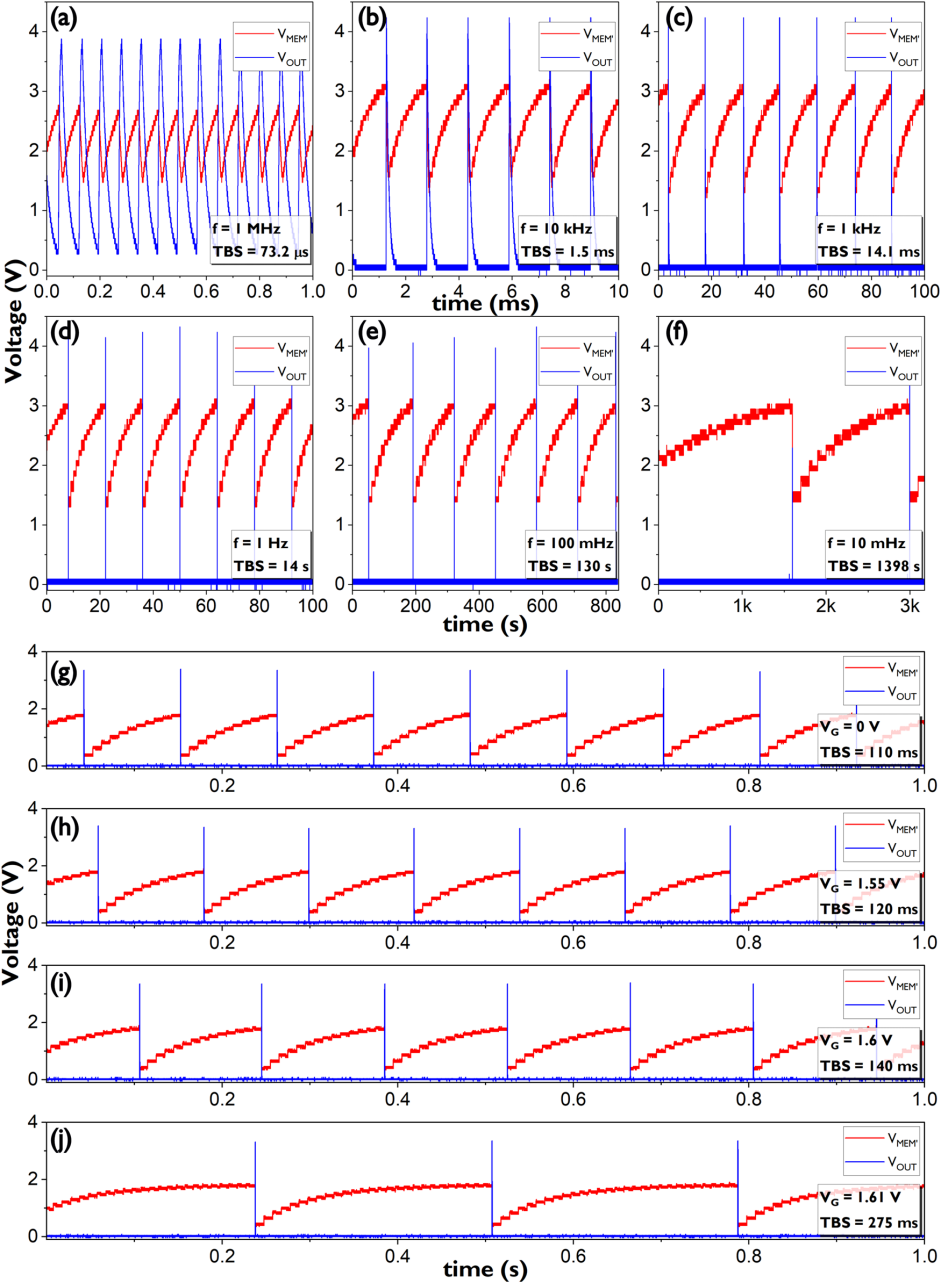
Multi-timescale TFT neuron wide range of time between spikes (TBS). **a**–**f** TBS range for minimum membrane permeability and various synapse pulse train frequencies. **g**–**j** Additional TBS values generated by varying membrane permeability (via the voltage *V*_G_) for a fixed synapse pulse train (100 Hz). Here, one can see that *q*_syn_ depends on instantaneous *V*_MEM_: the lower *V*_MEM_, the higher the upward step. Additionally for *V*_G_ = 1.6 V it is visible that the slope of *V*_MEM’_ is positive when *V*_MEM’_ < 1 V. This is because the synapse transistor is leaking for this value of *V*_G_ and overcoming the leak of the 100 times larger TFT modelling the membrane’s permeability. *V*_MEM’_ is the output of the source follower (SF) following the membrane potential *V*_MEM_ and *V*_OUT_ is the output of the neuron buffered by an additional SF circuit, see Fig. 4. TFT Thin-Film Transistor.

A set of outputs of the multi-timescale TFT neuron where the membrane permeability is set to the minimum by completely turning off the leakage TFTs in parallel with *C*_MEM_ and where the input frequency of the pulse train in the synapse is varied can be seen in Fig. 5a–f. The TBS of the spikes ranges from 73.2 µs for a synapse pulse train of 1 MHz to 1398 s for a synapse pulse train of 10 mHz. This wide range of timescales in TBS is effectively over 7 orders of magnitude apart. As mentioned earlier, for a given pulse train frequency in the synapse, the membrane permeability can be modified using as little as one biasing voltage. In this case, Fig. 5g–j shows the different TBS obtained for different *V*_G_ voltages with a fixed synapse pulse train with frequency of 100 Hz. Variations in membrane permeability for said frequency generated TBS values from 110 ms up to 275 ms for a *V*_G_ sweep range of 1.61 V.

**Fig. 6:**
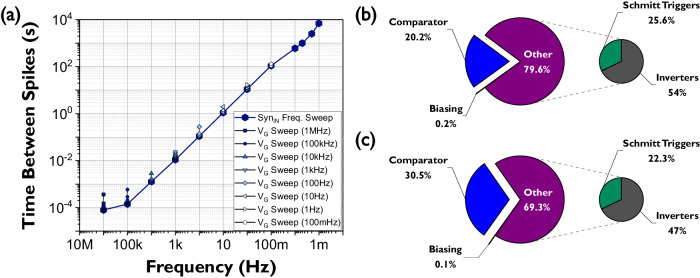
Multi-timescale TFT neuron full TBS range and power consumption breakdown. **a** Neuron’s adaptation to the level of activity at the synapse for *V*_G_ = −1 V (dark dots) and additional TBS ranges corresponding to changes in the membrane’s permeability for fixed levels of synapse activity (light dots). **b** Power consumption breakdown for *V*_DD_ = 5 V (74 µW total) for all synapse pulse train frequencies except 1 MHz (**c**). Power consumption breakdown for *V*_DD_ = 5 V (85 µW total) for the synapse input train frequency of 1 MHz. TFT Thin-Film Transistor. TBS Time Between Spikes.

The total range of TBS timescales attainable by the multi-timescale TFT neuron is present in Fig. 6a where both the full 7-orders-of-magnitude range corresponding to the neuron’s adaptation to the level of activity at the synapse and the additional TBS ranges corresponding to changes in the membrane’s permeability for a fixed level of activity are displayed. The multi-timescale TFT neuron has the potential to be a low power circuit due to the fact that it is a mostly idle circuit. However, the are several challenges expected when using an IGZO stack such as just having an N-type device available. Due to the resistive design approach taken to increase the gain of certain stages the total power consumption of the full circuit is 74 µW. The static power consumption is dominant since the only point where the circuit will consume more power is when a spike is generated. The spike time is very short compared to the idle periods (1.4 × 10^-6^ % for the longest TBS). Power consumption increases to 85 µW only at the highest excitation frequency of 1 MHz since the idle period shortens and becomes comparable to the spike width. A power breakdown for minimum membrane permeability is given in Fig. 6b, c, and a comparison with other state-of-the-art neurons is presented in the discussion section. The multi-timescale TFT neuron measures an area of 460 µm by 254 µm from which 48% is occupied by the TFTs modelling membrane permeability, see Fig. 4b. Both the power and area can be further improved, the former by optimizing the resistive parts of the design and the latter by reducing the size and number of TFTs modelling the permeability. These optimisations would reduce power and simultaneously increase the timescale range of the circuit to values beyond the 7-orders-of-magnitude range such as the 10-order-of-magnitude oscillations presented in this work’s supplementary material along with IGZO’s future challenges for the design of neuromorphic circuits. Migration towards CMOS back-end-of-line (BEOL) compatible TFT stacks such the one presented by Belmonte et al.^50^. would also improve metrics such as power and area; this migration would also take TFT neuromorphic circuits one step closer to full integration with CMOS technology. All these optimizations will be addressed in detail in the discussion section.

### Spiking Neural Network simulations

**Fig. 7:**
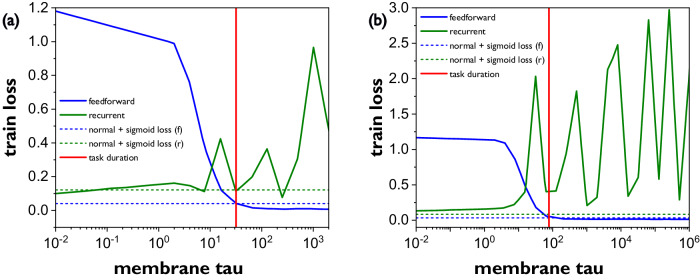
Addition task based of the Long Short-Term Memory (LSTM) principle. The time between spikes that exercise the long-term memory (and addition result) have been randomized over a number of trials for **a** a maximum task duration of 40 timesteps, displaying the prediction error against membrane’s time constant of the neurons for a given SNN either recurrent (r) or feedforward (f), and **b** a maximum task duration of 100 timesteps. The feedforward network has lower error (or best memory) with longer time constants as it can remember the sequences where the operands are far apart, while the recurrent network needs shorter time constants to forget noise amplified by reinforcement of memory from the recurrencies. The membrane tau values here presented can be comfortably covered by our neuron as can be seen in Fig. 5 and Fig. 6. SNN Spiking Neural Network.

Once the real capabilities of IGZO TFTs in the context of a neuron circuit were extracted, algorithm experiments to confirm the impact of tunable wide-range multi-timescale neurons were performed based on the well-known addition task benchmark that was introduced for demonstrating the Long Short-Term Memory (LSTM), by Hochreiter et al.^52^. In this task two inputs are provided to a network, one being a random number sequence and the other a control sequence that contains mostly (near) zero values apart from two time points when a distinct pulse (spike) is triggered. The goal of the network is to add the two numbers from the random sequence at the time points where the pulses were triggered by the control signal. As the time distance between the pulses increases the ability of the network to maintain long-term memory is put to test. For feed-forward networks of stateful neurons their ability to fulfil the task depends exclusively on the membrane time constant as a modulator of the neurons’ long-term memory. For recurrent networks their ability to maintain long-term memory depends on the combination of the membrane time constant and the strength of recurrent (feedback) synapses. Figure 7 provides more details about this study by plotting the error of two SNNs (one recurrent and one feed-forward) against the membrane time constant (tau) of output layer neurons averaged over a number of trials. In this study the time between the spikes that exercise the long-term memory (and addition result) has been varied randomly for total task durations of 40 and 100 timesteps. The plots show that the recurrent network has low intrinsic losses (good performance) for short values of tau up to a value that relates to the total task duration. As the memory of its neurons increases, they also integrate system noise that can be retained and amplified (due to recurrent feedback) for longer periods causing the performance of the network to be unstable at larger timeframes and thus making such a configuration unsuitable for tasks of long durations. On the other hand, for shorter time constants (tau), the recurrent neurons yield a satisfactory quality of memory where the recurrencies are mainly responsible for reinforcing the memory and the time constant plays the role of regulating dynamically the amplifying effect of recurrencies for the noise (Supplementary Fig. 15). The feedforward SNN on the other hand, shows the performance improving with increasing membrane time constant, and reaching optimal memory quality when the membrane’s time constant is comparable or longer than the task duration. The neurons in a feedforward network tend to forget easily since there is no state reinforcement from explicit recurrency, and opposite to the recurrent SNN case, the slower the membrane decays the longer memory is preserved. In other words, the model benefits from longer time constants for slow information integration (Supplementary Fig. 16). The horizontal dashed lines in the plots show the reference performance attained when the time-constants were subjected to training, as parameters (i.e. they can attain multiple different values instead of being fixed across all neurons). In summary, depending on the duration of the task at hand, and the SNN structure, the optimal time constants will vary, and we see that we can a-priori assert that the optimal time constant is relative to the task duration; longer tasks suggest longer optimal time constants. Moreover, depending on the different timescales present in the task and the structure of the network we have concrete cues for setting upper or lower bounds when fixing them as constants in the model or for initializing them when having them as learnable parameters to avoid local optima during training. These experiments confirm our intuition that systems can truly benefit from the programmable and wide range of timescales that are made viable by utilizing IGZO TFTs’ leakage currents to control the timescales of a neuron. Finally, the ability to use simpler feedforward neuron models empowered with long-term memory through large enough time-constants, enables more memory and area-efficient neural models. This efficiency can be fully exploited in a hybrid neuron circuit where IGZO TFTs are used to complement the already efficient CMOS neurons.

## Discussion

The multi-timescale TFT neuron presented in this work is a first step towards hybrid neuron circuits. Our findings prove the utility and versatility that an IGZO TFT technology can add to neuron circuits and aims to complement existing neuromorphic solutions both in CMOS and NVM technology. We believe that not one single technology but the combination of several is the path forward towards high performance multi-timescale spiking neuromorphic hardware. The ideal multi-timescale neuron would be one drawing strength from (at least) both CMOS and IGZO TFTs. Nevertheless, a first order comparison can still be made (as an indication) in terms of firing rate (in other words 1/TBS) and energy consumption per spike using the results from our neuron. Table 1 compares state-of-the-art solutions in CMOS, NVM and thin-film technologies. One of the key aspects of this paper is the firing rate, where Si CMOS and NVM can offer rates down to Hz range, TFTs show sub-Hz opportunities enabling SNN solutions for bio signals and other applications requiring a wide range of bio-compatible timescales. Our work shows 7 orders of magnitude firing range ratio (1.9 × 10^7^) between the fastest and the slowest TBS values, starting at tens of kHz down to even µHz and suggests at an even wider range of timescales of up to 10 orders of magnitude (CCO with ladder circuit).

**Table 1:** Comparison of this work to state-of-the-art solutions in CMOS, NVM and thin-film technologies

Reference	^17^	^38^	^61^	^62^	^28^	^27^	^46^	^47^	This work
Tech.	CMOS 180 nm	CMOS 28 nm	CMOS 180 nm	CMOS 28 nm	CMOS 22 nm	NVM	OFET 30 µm	IGZO 5 µm	IGZO 0.8 µm
Type	Mixed	Mixed	Mixed	Digital	Mixed	Simulation	Analogue	Analogue	Analogue
V_DD_	1.8 V	0.7 V–1 V	1.8 V	0.77 V	0.8 V	0.5 V–3 V	4 V	5 V	5 V
En. /Spike	883 pJ	2.3 nJ–30 nJ	10 pJ	800 pJ	990 fJ	^e^1–1000 e	^f^40 µJ	^h^10 nJ –160 nJ	103 mJ – 6.2 nJ
^a^Fire rate (FR)	Hz	kHz–Hz	kHz–Hz	Hz	kHz–Hz	MHz–Hz	Hz–mHz	^h^Hz–mHz	kHz–µHz
FR Dyn. Range	NA	3	3	NA	3	3	3	3	7
Area	^b^135 µm^2^	^c^1664 µm^2^	2025 µm^2^	14.3 µm^2^	^d^1799 µm^2^	Not fabricated	^g^2.25 mm^2^	0.28 mm^2^	0.106 mm^2^

^a^ Fire rate = 1/ TBS.

^b^ Estimation for 1 neuron and 64 synapse connections. 22.8% of the total area (38.5 mm^2^) was used for all 1 k neurons and 64k synapses.

^c^ 128 synapse connections of 13 µm^2^ each, output neuron area not available.

^d^ Only size estimation of the capacitors is available.

^e^ Electrons to spike.

^f^ En. /Spike = (power measured x measurement time ∆t)/ (spikes in ∆t).

^g^ Estimation from micrograph.

^h^ TFT neuron without refractory period modulation.

*CMOS* Complementary Metal-Oxide Semiconductor, *NVM* Non-Volatile Memory, *OFET* Organic Field-Effect Transistor, *IGZO* Indium-Gallium-Zinc-Oxide, *TBS* Time Between Spikes.

The energy consumption of the TFT neuron can be as low as nJ, but monopolar n-type only design comes with expected challenges such as an increased static energy consumption irrespective of the firing rate, which can be problematic when the number of neurons is greatly increased. Consequently, future work will aim to reduce this energy consumption to pJ or even lower levels when targeting SNN calculations in hardware on edge-type devices. Low Temperature Polysilicon (LTPS) is a complementary (p-type and n-type) thin-film technology that is combined with IGZO TFTs to create a technology called Low Temperature Polysilicon Oxide (LTPO)^53,54^. If one wished the neuron to be fully implemented in thin-film technology, the availability of complementary devices makes LTPO a great option to largely reduce the static energy consumption of the neuron while keeping the timescale control dependent on IGZO TFTs. In this case, one must make sure the TFTs modelling membrane permeability remain IGZO TFTs while other structures in the neuron, such as the inverters and comparator, can capitalize on the low static energy of complementary design. Çeliker et al.^55^ have demonstrated a power reduction for a microprocessor manufactured in either IGZO or LTPS. Nevertheless, we believe the full potential of IGZO TFTs in neuromorphic circuits will be reached when combined with silicon CMOS technologies. Thus, another possible route to reduce power consumption, and the intended direction of our research, is to design a CMOS neuron and control (at least) the neuron’s permeability using IGZO TFTs. The ideal scenario, even further into the future, would be combining CMOS silicon wafers with BEOL compatible IGZO TFTs which would not only reduce energy consumption but also greatly reduce the area of the final neuron as BEOL IGZO TFTs have gate lengths in the range of tens of nanometers^44,50^ and do not share real state with CMOS transistors since those are located in front-end-of-line (FEOL) layers. The combination of BEOL IGZO TFTs and FEOL CMOS should also keep wire lengths short even for large numbers of neurons, which is important both for delay and energy overhead. Kumari et al.^40^ not only highlight the positive impact of having control over the available timescales generated by an integrate and fire neuron but also demonstrate this can be done in a compact area by making a design in CMOS technology, albeit only in simulation.

It is known for IGZO as semiconductor that thermal and bias effects may impact its threshold voltage^44,56^. However, in those works, the low-level leakage currents are maintained, while a shift in threshold voltage is observed. In our proposed circuit, this shift in threshold voltage will indeed impact the behaviour of the neuron and will reduce the dynamic range of the firing rate when the bias voltage is selected close to the subthreshold region. Moreover, bias stress effects become worse with large gate fields and thus gate-source voltages, which should be kept as low as possible. Therefore, *V*_G_ and *V*_TFTleak_ of the IGZO transistor should be kept as low as possible, preferably around −1 V to −2 V, resulting in only a very small shift in threshold voltage to preserve the correct operation of the neuron. Furthermore, IGZO TFTs’ reliability and threshold voltage shift may also be improved via material and device engineering^57–60^.

The TFTs modelling neuron permeability can be reduced in number and size. The current configuration was chosen so that the generated timescales could be properly measured with the tools at our disposal, these conditions defined our choice to utilize 100 TFTs to model membrane permeability. Future work aims at modelling the neuron’s permeability using only one IGZO TFT preferably of minimum size.

## Conclusion

This work exploits the benefits of the ultra-low leakage currents from IGZO TFTs in the context of analogue spiking neurons in SNNs and through this aims to direct research efforts towards hybrid systems where not only CMOS and NVM but also IGZO is present. Once the IGZO ultra-low leakage currents for a particular thin-film technology stack are known, they can be harnessed and utilized to create a multi-timescale neuron (in this work and as a first step a full TFT neuron) capable of generating a wide space of analogue timescales in between spikes. The designed multi-timescale TFT neuron exhibits a broad range of timescales over 7 orders of magnitude long in time between spikes which is one of the longest reported for a spiking neuron circuit and possibly the slowest (715 µHz) firing rate to date, to the author’s best knowledge. This fully configurable neuron is extremely versatile as it will introduce different timescales into an SNN just by reacting to the excitation strength at its synapse. A simple biasing voltage further adds flexibility in adjusting or changing these timescales. Power consumption is currently a challenge and can be reduced by combining IGZO TFTs with other technologies such as LTPO in thin-film or CMOS in silicon wafers. The timescale range enabled by IGZO TFTs not only adds heterogeneity to SNNs increasing their performance but can also compensate for silicon CMOS’ and NVM’s weaknesses to cost-effectively support this wide range of analogue timescales. The latter paves the way towards CMOS, NVM and TFT hybrid solutions in which one offsets the deficiencies of the others. The combination of the best traits in all three technologies will promote the realization of simpler SNN hardware with bio-compatible timescales capable of solving increasingly complex tasks for prolonged periods of time without having to draw upon additional algorithms or using costly real estate in CMOS technology unless strictly necessary.

## Methods

### Atto-ampere current circuit simulations

Design and simulations of all low current circuits were based on the model and baseline PDK provided by the IGZO-foundry Pragmatic. Atto-ampere simulations are very challenging and require careful manipulation of the convergence aids of the simulation tool utilized, in this case Cadence Virtuoso. Interpretation of the results must also be handled with care as these extremely low current levels can lead to simulations results that may not have a reasonable physical interpretation or that might not accurately describe real results.

### Measurement of all timescales

All measurements were captured with a PC oscilloscope PicoScope model 3406DMSO and Pico TA132 probes. This oscilloscope was selected due to its ability to accommodate large measurement times without “rolling” and because it can save the large files generated by these long measurement times with sampling rates that were several orders of magnitude faster directly on the PC. The results on all neuron-related figures are the outcome of the best compromise between required measurement time, sampling rate and file size. The main challenges (in resolution) were encountered at the slower excitation frequencies since the neuron’s spikes remained in the order of micro-seconds while the measurement time elongated for hours/days. Thus, the optimal sampling rate for an expected file size was set. Slightly limiting the sampling rate resulted in measurement artifacts that sometimes show neuron spikes of different amplitudes specially for cases with the longest measurements times. Proper spiking can also be followed via the membrane voltage. In addition, to aid discharging the very large capacitance of the PicoScope probes, a smaller 100 kΩ external resistor was added in parallel with the probes, this resistor dictates the discharge speed whose influence can be seen in faster spiking measurements but also reduced the spike amplitude in the slower measurements. The resistor was kept the same for all measurements.

### Algorithm experiments

A set of LIF-based SNN simulations were performed in PyTorch. The input data was a two-dimensional sequence where one channel streams random uniform values in [0,1], and the second channel streams zeros except at two randomly selected times, for every half of the first 80% of the task duration, where the streamed value is one. The input channels are fully connected to a hidden layer of 64 neurons with firing threshold, which are in turn fully connected to a readout neuron without firing threshold. The target (the dot product of both input channels) is compared to the average readout neuron’s membrane potential on the last 20% of the sequence, using the Mean Squared Error. A batch size of 128 (as presented by Hochreiter et al.^52^) is chosen and the network is trained for 1000 epochs, using BPTT with surrogate-gradient learning. Recurrent and feedforward configurations for the hidden layer were trained, with each model using homogeneous time constants in the range [0.01, 20,…, 211], yielding a total of 26 trained models for a given sequence length. The reported error is an average of 100 batches not seen in training. The training was performed in an NVIDIA GeForce RTX 3060 GPU.

## Supplementary information

Supplementary Information

## Data Availability

Data from the current study is available from the corresponding author on reasonable request.

## Code availability

The code used in the SNN simulation is available from the corresponding author on reasonable request.

## References

[1] Geng, D. et al. Thin-film transistors for large-area electronics. *Nat. Electron.* **6**, 963–972 (2023).

[2] Portilla, L. et al. Wirelessly powered large-area electronics for the Internet of Things. *Nat. Electron.* **6**, 10–17 (2023).

[3] Celiker, H., Sou, A., Cobb, B., Dehaene, W. & Myny, K. Flex6502: A Flexible 8b Microprocessor in 0.8 µm metal-oxide thin-film transistor technology implemented with a complete digital design flow running complex assembly code. In *2022 IEEE International Solid-State Circuits Conference (ISSCC).* 272–274 (IEEE, 2022).

[4] Biggs, J. et al. A natively flexible 32-bit Arm microprocessor. *Nature* **595**, 532–536 (2021). 34290427 10.1038/s41586-021-03625-w

[5] Papadopoulos, N. et al. Touchscreen tags based on thin-film electronics for the Internet of everything. *Nat Electron* **2**, 606–611 (2019). 31872176 10.1038/s41928-019-0333-z PMC6927798

[6] Papadopoulos, N., Lopez, M. V., Ameys, M., Huang, T. C. & Myny, K. 11-4: 3.55-watt output power LTPS TFT DCDC converter for actuators on wearable devices on flexible substrate. *SID Symp. Dig.Tech. Pap.* **54**, 132–135 (2023).

[7] Wang, C. et al. Continuous monitoring of deep-tissue haemodynamics with stretchable ultrasonic phased arrays. *Nat. Biomed. Eng.* **5**, 749–758 (2021). 34272524 10.1038/s41551-021-00763-4

[8] Sim, K. et al. An epicardial bioelectronic patch made from soft rubbery materials and capable of spatiotemporal mapping of electrophysiological activity. *Nat. Electron.* **3**, 775–784 (2020).

[9] Kim, Y. et al. A bioinspired flexible organic artificial afferent nerve. *Science (1979)* **360**, 998–1003 (2018). 10.1126/science.aao0098 29853682

[10] Wu, C., Kim, T. W., Choi, H. Y., Strukov, D. B. & Yang, J. J. Flexible three-dimensional artificial synapse networks with correlated learning and trainable memory capability. *Nat. Commun.* **8**, 752 (2017). 10.1038/s41467-017-00803-1 PMC5622032 28963546

[11] Ozer, E. et al. A hardwired machine learning processing engine fabricated with submicron metal-oxide thin-film transistors on a flexible substrate. *Nat. Electron.* **3**, 419–425 (2020).

[12] Nambiar, V. P. et al. 0.5V 4.8 pJ/SOP 0.93μW Leakage/core neuromorphic processor with asynchronous NoC and reconfigurable LIF neuron. In *2020 IEEE Asian Solid-State Circuits Conference, A-SSCC 2020* (ed. Jung, w. et al.) 1–4 (Institute of Electrical and Electronics Engineers Inc., 2020).

[13] Frenkel, C., Legat, J.-D. & Bol, D. MorphIC: A 65-nm 738k-synapse/mm$^2$ quad-core binary-weight digital neuromorphic processor with stochastic spike-driven online learning. *IEEE Trans. Biomed. Circuits Syst.* **13**, 999–1010 (2019). 31329562 10.1109/TBCAS.2019.2928793

[14] Pei, J. et al. Towards artificial general intelligence with hybrid tianjic chip architecture. *Nature* **572**, 106–111 (2019). 31367028 10.1038/s41586-019-1424-8

[15] Davies, M. et al. Loihi: A neuromorphic manycore processor with on-chip learning. *IEEE Micro.* **38**, 82–99 (2018).

[16] Basu, A., Deng, L., Frenkel, C. & Zhang, X. Spiking neural network integrated circuits: a review of trends and future directions. In *2022 IEEE Custom Integrated Circuits Conference (CICC)* (eds. Luo, x. et al.) 1–8 (IEEE, 2022).

[17] Moradi, S., Qiao, N., Stefanini, F. & Indiveri, G. A scalable multicore architecture with heterogeneous memory structures for dynamic neuromorphic asynchronous processors (DYNAPs). *IEEE Trans. Biomed. Circuits Syst.* **12**, 106–122 (2018). 29377800 10.1109/TBCAS.2017.2759700

[18] Wan, W. et al. 33.1 A 74 TMACS/W CMOS-RRAM neurosynaptic core with dynamically reconfigurable dataflow and in-situ transposable weights for probabilistic graphical models. In *2020 IEEE International Solid-State Circuits Conference—(ISSCC)* 498–500 (IEEE, 2020).

[19] Schemmel, J., Billaudelle, S., Dauer, P. & Weis, J. Accelerated analog neuromorphic computing. In *Analog Circuits for Machine Learning, Current/Voltage/Temperature Sensors, and High-speed Communication* (eds. Harpe, P., Makinwa, K. A., Baschirotto, A.) 83–102 (Springer, 2020).

[20] Huynh, P. K. et al. Implementing spiking neural networks on neuromorphic architectures: a review. 10.48550/arXiv.2202.08897 (2022).

[21] Azghadi, M. R. et al. Hardware implementation of deep network accelerators towards healthcare and biomedical applications. *IEEE Trans. Biomed. Circuits Syst.* **14**, 1138–1159 (2020). 33156792 10.1109/TBCAS.2020.3036081

[22] Burelo, K. et al. A spiking neural network (SNN) for detecting high frequency oscillations (HFOs) in the intraoperative ECoG. *Sci. Rep.* **11**, 6719 (2021). 10.1038/s41598-021-85827-w PMC7990937 33762590

[23] Jaeger, H. & Catthoor, F. Timescales: the choreography of classical and unconventional computing. 10.48550/arXiv.2301.00893 (2023).

[24] Lukoševičius, M., Popovici, D., Jaeger, H. & Siewert, U. *Time Warping Invariant Echo State Networks*. https://www.ai.rug.nl/minds/uploads/techreport2.pdf (2006). 10.1016/j.neunet.2007.04.016 17517495

[25] Xia, Q. & Yang, J. J. Memristive crossbar arrays for brain-inspired computing. *Nat. Mater* **18**, 309–323 (2019). 30894760 10.1038/s41563-019-0291-x

[26] Chakraborty, I., Jaiswal, A., Saha, A. K., Gupta, S. K. & Roy, K. Pathways to efficient neuromorphic computing with non-volatile memory technologies. *Appl. Phys. Rev.* **7**, 021308 (2020).

[27] Goda, A., Matsui, C. & Takeuchi, K. A stochastic leaky-integrate-and-fire neuron model with floating gate-based technology for fast and accurate population coding. *IEEE*. *J. Electron. Devices Soc.* **10**, 861–869 (2022).

[28] Rubino, A., Payvand, M. & Indiveri, G. Ultra-low power silicon neuron circuit for extreme-edge neuromorphic intelligence. In *2019 26th IEEE International Conference on Electronics, Circuits and Systems (ICECS)* 458–461 (IEEE, Genoa, Italy, 2019).

[29] Linares-Barranco, B. & Serrano-Gotarredona, T. On the design and characterization of femtoampere current-mode circuits. *IEEE J. Solid-State Circuits* **38**, 1353–1363 (2003).

[30] He, X., Liu, T., Hadaeghi, F. & Jaeger, H. Reservoir transfer on analog neuromorphic hardware. In *2019 9th International IEEE/EMBS Conference on Neural Engineering (NER).* 1234–1238 (IEEE, 2019).

[31] Perez-Nieves, N., Leung, V. C. H., Dragotti, P. L. & Goodman, D. F. M. Neural heterogeneity promotes robust learning. *Nat. Commun.* **12**, 5791 (2021). 10.1038/s41467-021-26022-3 PMC8490404 34608134

[32] Payvand, M. et al. Self-organization of an inhomogeneous memristive hardware for sequence learning. *Nat. Commun.* **13**, 5793 (2022). 36184665 10.1038/s41467-022-33476-6 PMC9527242

[33] Patiño-Saucedo, A. et al. Empirical study on the efficiency of spiking neural networks with axonal delays, and algorithm-hardware benchmarking. In *2023 IEEE International Symposium on Circuits and Systems (ISCAS).* 1–5 (IEEE, 2023).

[34] Kang, W. M. et al. A spiking neural network with a global self-controller for unsupervised learning based on spike-timing-dependent plasticity using flash memory synaptic devices. in *2019 International Joint Conference on Neural Networks (IJCNN)* 1–7 (IEEE, 2019).

[35] Cantley, K. D., Subramaniam, A., Stiegler, H. J., Chapman, R. A. & Vogel, E. M. Hebbian learning in spiking neural networks with nanocrystalline silicon TFTs and memristive synapses. *IEEE Trans. Nanotechnol.* **10**, 1066–1073 (2011).

[36] Arthur, J. V. & Boahen, K. A. Silicon-neuron design: a dynamical systems approach. *IEEE Trans. Circuits Syst. I: Regular Pap.* **58**, 1034–1043 (2011). 10.1109/TCSI.2010.2089556 PMC3100558 21617741

[37] Indiveri, G. et al. Neuromorphic silicon neuron circuits. *Front. Neurosci.* **5**, 9202 (2011). 10.3389/fnins.2011.00073 PMC3130465 21747754

[38] Mayr, C. et al. A biological-realtime neuromorphic system in 28 nm CMOS using low-leakage switched capacitor circuits. *IEEE Trans. Biomed. Circuits Syst.* **10**, 243–254 (2016). 25680215 10.1109/TBCAS.2014.2379294

[39] Wijekoon, J. H. B. & Dudek, P. A CMOS circuit implementation of a spiking neuron with bursting and adaptation on a biological timescale. In *2009 IEEE Biomedical Circuits and Systems Conference* 193–196 (IEEE, 2009).

[40] Shah, A. K., Cho, E. S., Park, J., Shin, H. & Cho, S. A compact integrate-and-fire neuron circuit embedding operational transconductance amplifier for fidelity enhancement. *IEEE Access* **11**, 53932–53938 (2023).

[41] Serrano-Gotarredona, T. & Linares-Barranco, B. 7-decade tuning range CMOS OTA-C sinusoidal VCO. *Electron Lett.* **34**, 1621–1622 (1998).

[42] Linares-Barranco, B., Sanchez-Sinencio, E., Newcomb, R. W., Rodriguez-Vazquez, A. & Huertas, J. L. Novel CMOS analog neural oscillator cell. *Proc. IEEE Int. Symp. Circuits Syst.* **2**, 794–797 (1989).

[43] Sekine, Y. et al. Invited success in measurement the lowest off-state current of trnsistor in the world. *ECS Trans.* 37, 77–88 (2019). .

[44] Kunitake, H. et al. High thermal tolerance of 25-nm c-axis aligned crystalline In-Ga-Zn oxide FET. In *2018 IEEE International Electron Devices Meeting (IEDM).* 13.6.1−13.6.4 (IEEE, 2018).

[45] Jiang, C. et al. Mammalian-brain-inspired neuromorphic motion-cognition nerve achieves cross-modal perceptual enhancement. *Nat. Commun.* **14**, 1344 (2023). 36906637 10.1038/s41467-023-36935-w PMC10008641

[46] Mirshojaeian Hosseini, M. J. et al. Organic electronics axon-hillock neuromorphic circuit: towards biologically compatible, and physically flexible, integrate-and-fire spiking neural networks. *J. Phys. D Appl. Phys.* **54**, 104004 (2021).

[47] Lebanov, A. et al. Flexible unipolar IGZO transistor-based integrate and fire neurons for spiking neuromorphic applications. *IEEE Trans. Biomed. Circuits Syst.* **18**, 200–214 (2024). 37782619 10.1109/TBCAS.2023.3321506

[48] Steudel, S. et al. Power saving through state retention in IGZO-TFT AMOLED displays for wearable applications. *J. Soc. Inf. Disp.* **25**, 222–228 (2017).

[49] Ishizu, T. et al. A 48 MHz 880 nW standby power normally-off MCU with 1 clock full backup and 4.69 μs wakeup featuring 60 nm crystalline In–Ga–Zn oxide BEOL-FETs. In *2019 Symposium on VLSI Circuits* C48–C48 (IEEE, Kyoto, 2019).

[50] Belmonte, A. et al. Tailoring IGZO-TFT architecture for capacitorless DRAM, demonstrating > 103s retention, >1011cycles endurance and lgscalability down to 14 nm. In *Technical Digest—International Electron Devices Meeting, IEDM.*10.6.1–10.6.4 (IEEE, 2021).

[51] Celiker, H., Sou, A., Dehaene, W. & Myny, K. Two-stage resistor-load logic for digital applications on flexible substrates. In *2021 IEEE International Conference on Flexible and Printable Sensors and Systems (FLEPS)* 1–4 (IEEE, 2021).

[52] Hochreiter, S. & Schmidhuber, J. Long short-term memory. *Neural Comput.* **9**, 1735–1780 (1997). 9377276 10.1162/neco.1997.9.8.1735

[53] Chang, T. K., Lin, C. W. & Chang, S. 39-3: Invited paper: LTPO TFT technology for AMOLEDs†. *SID Symp. Digest Tech. Pap.* **50**, 545–548 (2019).

[54] An, J. et al. Gate driver on array with multiple outputs and variable pulse widths for low-temperature polysilicon and oxide (LTPO) TFTs driven AMOLED displays. *IEEE Trans. Circuits Syst. II: Express Briefs* **70**, 934–938 (2023).

[55] Çeliker, H., Dehaene, W. & Myny, K. Multi-project wafers for flexible thin-film electronics by independent foundries. *Nature* **629**, 335–340 (2024). 38658759 10.1038/s41586-024-07306-2 PMC11078730

[56] Chiu, J. C. et al. Performance Improvement by Double-Layer a-IGZO TFTs with a Top Barrier. *IEEE J. Electron. Devices Soc.* **10**, 45–50 (2022).

[57] Pan, Z. et al. Approaches to improve mobility and stability of IGZO TFTs: a brief review. *Trans. Electr. Electron. Mater.*10.1007/s42341-024-00536−1 (2024).

[58] Liu, W.-S., Hsu, C.-H., Jiang, Y., Lai, Y.-C. & Kuo, H.-C. Improving device characteristics of dual-gate IGZO thin-film transistors with Ar–O2 mixed plasma treatment and rapid thermal annealing. *Membranes (Basel)* **12**, 49 (2021). 35054574 10.3390/membranes12010049 PMC8780293

[59] Kim, J.-W. et al. Improvement in electrical stability of a-IGZO TFTs using thinner dual-layer dielectric film. *Metals (Basel)* **12**, 1663 (2022).

[60] Koretomo, D., Hamada, S., Mori, M., Magari, Y. & Furuta, M. Marked improvement in reliability of 150 °C processed IGZO thin-film transistors by applying hydrogenated IGZO as a channel material. *Appl. Phys. Express* **13**, 076501 (2020).

[61] Nair, M. V. & Indiveri, G. An ultra-low power sigma-delta neuron circuit. In *2019 IEEE International Symposium on Circuits and Systems (ISCAS)* 1–5 (IEEE, 2019).

[62] Merolla, P. A. et al. A million spiking-neuron integrated circuit with a scalable communication network and interface. *Science (1979)* **345**, 668–673 (2014). 10.1126/science.1254642 25104385

