# Author Correction: A tunable multi-timescale Indium-Gallium-Zinc-Oxide thin-film transistor neuron towards hybrid solutions for spiking neuromorphic applications

**DOI:** 10.1038/s44172-024-00336-8

**Published:** 2024-12-31

**Authors:** Mauricio Velazquez Lopez, Bernabe Linares-Barranco, Jua Lee, Hamidreza Erfanijazi, Alberto Patino-Saucedo, Manolis Sifalakis, Francky Catthoor, Kris Myny

**Affiliations:** 1 EPIC, Large Area Thin-film Transistor Electronics, imec, Kapeldreef 75, 3001 Leuven, Belgium; 2 ES&S, COSIC, ESAT, KU Leuven, 3590 Diepenbeek, Belgium; 3 Instituto de Microelectrónica de Sevilla, IMSE-CNM, (CSIC Universidad de Sevilla), 41092 Sevilla, Spain; 4 School of Information and Communication Engineering, Sungkyunkwan University (SKKU), 16419 Seoul, South Korea; 5 Imec The Netherlands, High-Tech Campus 31, 5656 Eindhoven, The Netherlands; 6 ESAT, KU Leuven, 3000 Leuven, Belgium

**Keywords:** Electrical and electronic engineering, Electronics, photonics and device physics, Nanoscience and technology

**Correction to:** *Communications Engineering* 10.1038/s44172-024-00248-7, published online 23 July 2024

An individual who was originally acknowledged in our paper has requested that their acknowledgment be removed. This has now been corrected in both the PDF and html versions of the manuscript.

Therefore the updated Acknowledgements reads as:

“The authors thank Donald Raddoux from KU Leuven, Belgium for his help during the sample packaging efforts and François Berghmans from imec Leuven, Belgium for his contribution in additional measurements during the review process of this paper. The authors acknowledge the support from the Horizon 2020 European project MeM-Scales—Memory technologies with multi-scale time constants for neuromorphic architectures (grant agreement No. 871371). Part of this work has received funding under the Horizon Europe program from the European Research Council (ERC) under grant agreement No 101088591 “ORISON”. Views and opinions expressed are, however, those of the author(s) only and do not necessarily reflect those of the European Union or the European Research Council. Neither the European Union nor the granting authority can be held responsible for them. The authors would like to thank the foundry Pragmatic for fabricating the circuits presented in this paper.”

