# Supplementary material for: A tunable multi-timescale Indium-Gallium-Zinc-Oxide thin-film transistor neuron towards hybrid solutions for spiking neuromorphic applications
Source: Commun Eng. 2024 Jul 23;3:102. doi: 10.1038/s44172-024-00248-7 (PMC11266500; doi:10.1038/s44172-024-00248-7)
Supplement: Supplementary file 1 — Supplementary Information [file 44172_2024_248_MOESM1_ESM.pdf]

A tunable multi-timescale Indium-Gallium-Zinc-Oxide Thin-Film Transistor neuron towards hybrid solutions for spiking neuromorphic applications

Supplementary Materials

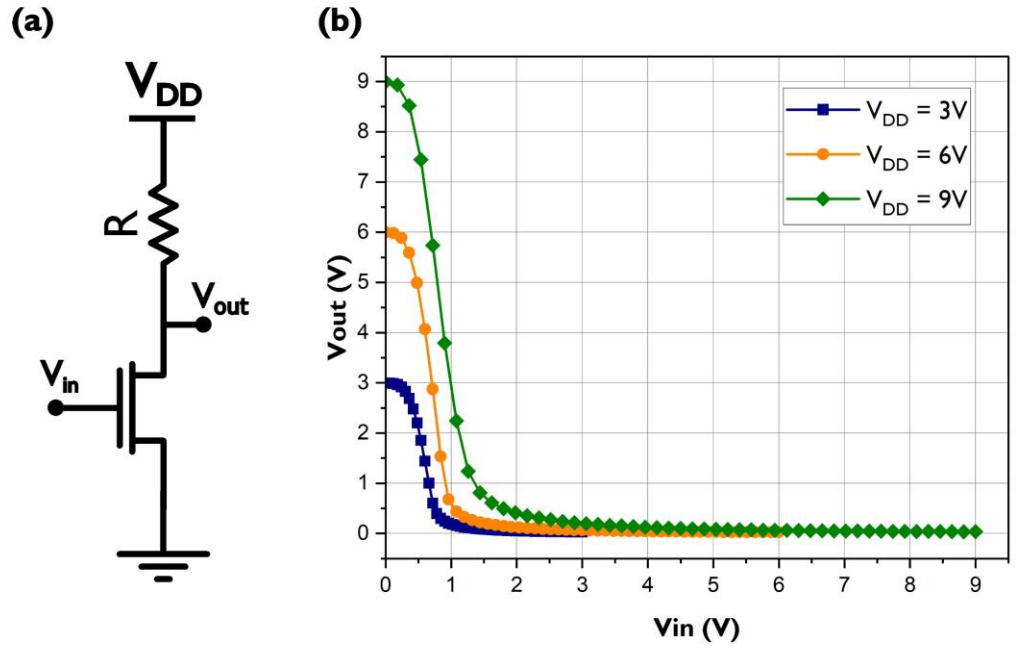

Supplementary Fig. 1. R-logic inverter. **a.** Circuit schematic. **b.** Measurements of transfer characteristics for different supply voltages showing the high gain and imbalance in tripping point.

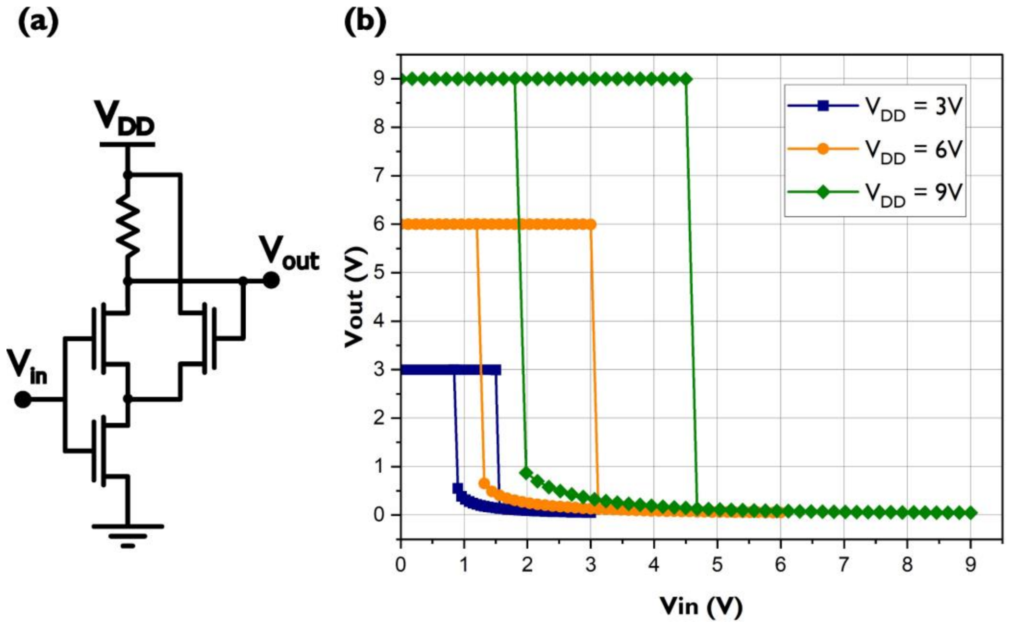

Supplementary Fig. 2. R-Schmitt trigger. **a.** Circuit schematic. **b.** Measurements of transfer characteristics showing the hysteresis window, which is fixed by the value of resistor R. The hysteresis window varies for different supply voltages.

Digitally-Controlled Ladder-Based Ultra-Wide Range Oscillator

In order to assess the potential of TFT technology to provide an ultra-wide range tuning capability by digitally adjusting a current source, we studied the viability of using a digitally-controlled transistor-based ladder structure to control the discharge current of a saw-tooth oscillator [jssc03].

Supplementary Fig. 3(a) shows the schematic of the sawtooth oscillator used. Transistor M_{reset}, when turned ON, resets capacitor C to voltage V_{TOP}. Once charged up, M_{reset} is turned OFF and capacitor C is discharged at a constant rate by digitally-controlled current source I_{leak} until $V_C = V_{BOTTOM}$, which is given by the left threshold of the hysteresis window shown in Supplementary Fig. 2, and which is power supply voltage dependent. At this point, the output of the hysteresis comparator V_{comp} changes from low to high, which after a delay δt, introduced by the delay element, turns ON M_{reset} and resets V_C to V_{TOP}. A hysteresis comparator is required because, when the capacitor discharge is extremely slow, the noise present at V_C would make V_{comp} to randomly change between high and low when approaching V_{BOTTOM}. The delay element is necessary to make sure transistor M_{reset} is turned ON during sufficient time to guarantee its ON resistance can charge up capacitor V_C to V_{TOP}.

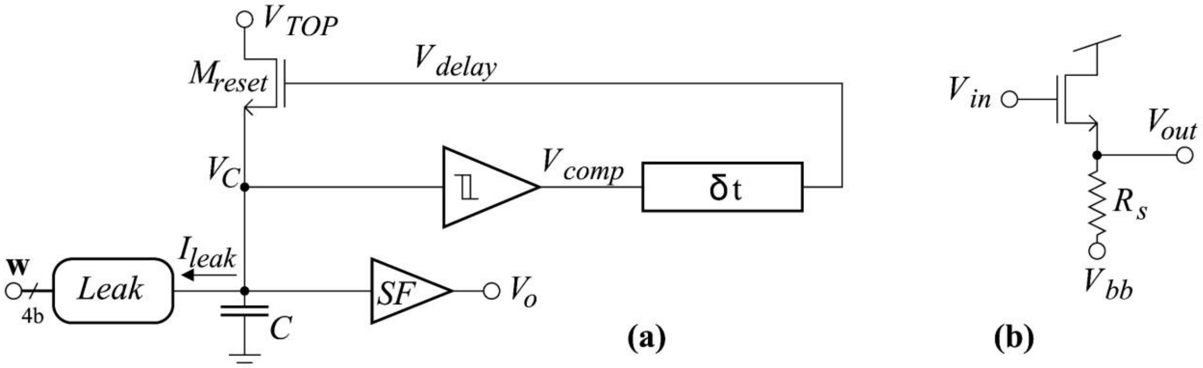

Supplementary Fig. 3. Ultra-wide range digitally-controlled sawtooth oscillator **a.** Simplified circuit schematics. **b.** Detail of source-follower circuit.

During normal capacitor discharge, transistor M_{reset} needs to be turned OFF completely, without any undesired leakage current, to guarantee it will not distort the characterization of the digitally-controlled leakage current source discharging capacitor C. For this, its V_{GS} voltage has to be set sufficiently negative, like $V_{GS} \leq -1.5V$. Transistor M_{reset} is turned OFF by setting its gate voltage $V_{delay} = 0V$. Consequently, its source voltage $V_C \geq 1.5V$. Since V_C will be oscillating between V_{TOP} and V_{BOTTOM}, one should set V_{DD} to guarantee $V_{BOTTOM} \geq 1.5V$.

In our measurements, we have typically set $V_{DD} = 6V$ (thus $V_{BOTTOM} \approx 1.5V$) and V_{TOP} between 3V and 4.5V. Voltage V_C is observed through the simple source-follower circuit shown in Supplementary Fig. 3(b). The nFET is on-chip, while the resistor is connected outside the chip. Ideally, a source follower would require a constant current source (instead of the resistor) to guarantee a constant gate-to-source voltage. Using a resistor will add a modulation to the gate-to-source voltage, making the source follower slightly non-linear. However, by setting V_{bb} sufficiently negative ($V_{bb} \leq -2V$) with $R_S \approx 100k\Omega$, its nonlinearity is minimized. This circuit will introduce a voltage shift of approximately 1V from input to output.

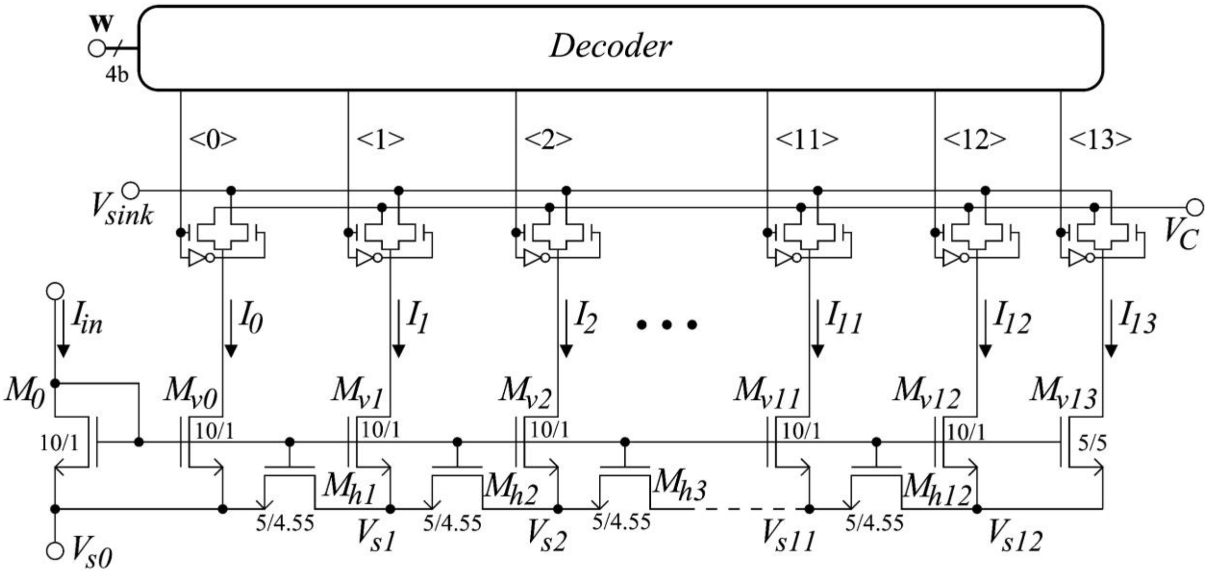

Supplementary Fig. 4. Digitally-controlled ladder-based leakage current source.

Supplementary Fig. 4 shows a 14-branch nFET ladder structure for current division [jssc03]. Vertical branch transistors M_{vj} are sized with W_{vj}/L_{vj} = N-1, horizontal branch transistors M_{hj} with W_{hj}/L_{hj} = N/(N-1), and the terminating transistor with W_{v13}/L_{v13} = 1. With this sizing, the current ratio between consecutive vertical branches would be

$$\frac{I_j}{I_{j+1}} = N \quad , \quad j = 0, \dots 11 \quad ; \quad \frac{I_{12}}{I_{13}} = N - 1$$

or, equivalently

$$I_j = \frac{I_{in}}{N^j} \quad , \quad j = 0, \dots 12$$

In our design, we chose N = 11, with W_{vj} = 10μm, L_{vj} = 1μm, W_{hj} = 5μm, L_{hj} = 4.55μm, for j = 0, … 12, and W_{v13} = L_{v13} = 5μm. We picked intentionally an excessive number of branches to reach the technology limit of minimum leakage currents. In practice, the last branches would drive the limit leakage current we want to find out.

The drains of the vertical transistors are connected to either a sinking node V_{sink} or to the capacitor node V_C, through digital control signals <*j*>. Only one branch will be connected to node V_C, while the rest will be connected to node V_{sink}. Lines <*j*> are set by a digital decoder, controlled by 4-bit digital word **w<0:3>**. Since we have picked N=11, ideally, we could select leakage currents scaling them down approximately one per decade. However, secondary effects will result in N > 11, as explained later.

The source voltages of the vertical transistors V_{sj} will be increasing smoothly from the minimum voltage set externally at V_{s0} to a maximum at V_{s12}. Voltage V_{s0} should be set low enough, so that V_{s12} guarantees a positive V_{DS} voltage at M_{v12} and M_{v13}. The minimum drain voltage is approximately V_{BOTTOM}. On the other hand, V_{s0} should not be too negative, to avoid too high V_{DS} voltages, which could stress the TFT transistors and degrade their very low leakage currents. We normally set V_{s0} between 0V and 2V.

For optimum current division between consecutive branches, it is desirable that $V_{sink} = V_C$. However, V_C will be oscillating between V_{TOP} and V_{BOTTOM}. As a compromise, we set $V_{sink} \approx (V_{TOP} + V_{BOTTOM})/2$.

The fact that source voltages V_{sj} increase with *j* makes that vertical transistors M_{vj}, which operate in saturation as current sources, will have larger V_{DS} voltages for smaller *j*, thus suffering from larger *Early Voltage Effect*. Therefore, for smaller *j*, vertical transistors will drive a slightly larger current than expected, leaving a smaller than expected current for the subsequent branches. This will have the secondary effect of resulting in a current ratio I_j/I_{j+1} greater than N.

Measurement results

Next, we show measurements on three sawtooth oscillator circuit samples. Measurements were performed by characterizing the discharging slopes of the saw-tooth oscillators. Supplementary Fig. 5 illustrates a measurement on oscillator sample Osc1 when activating branch <3>. The measured discharge slope is 117V/s. The corresponding discharge current we estimate by assuming capacitor in Supplementary Fig. 3 has a value of C = 1pF, resulting in a discharge current of 117pA.

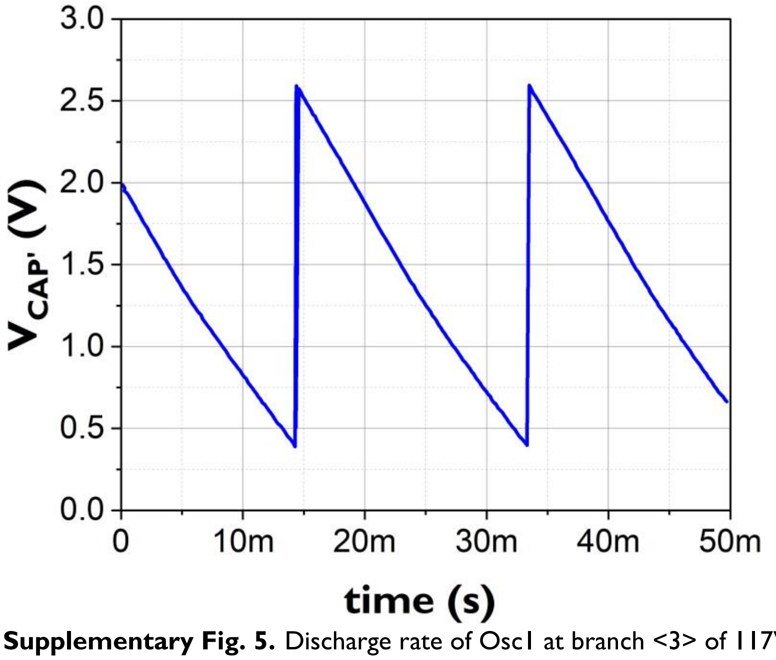

Supplementary Fig. 5. Discharge rate of Osc1 at branch <3> of 117V/s.

Collecting these measurements for the three oscillators we measured, results in the plots shown in Supplementary Fig. 6, where the measure discharge rate is shown on the left axis, while the inferred discharge current is shown on the right axis. We can see a similar behaviour for Osc2 and Osc3, for which the max ratio between consecutive current branches was 24 (between branches 4 and 5). However, the behaviour for Osc1 was slightly different, showing a max ratio between consecutive current branches of 295 (between branches 3 and 4). However, the max and min range was similar. The maximum spread was obtained for Osc2 with a ratio between max/min rates of $5e5Vs^{-1}/7.8e\text{-}6Vs^{-1} = 6.41 \times 10^{10}$, almost 11 decades!

Some other illustrative oscilloscope captures are shown in Supplementary Fig. 7-13. Note the extremely long measurement times required for branches <8> and <9> in supplementary Fig. 12-13, of more than 13 and 35 hours, respectively. The Osc1 branch<9> measurement is shown in Fig. 3d of the main text, where the discharge rate at its tail is 6.45uV/s, resulting in an estimated discharge current of 6.45aA.

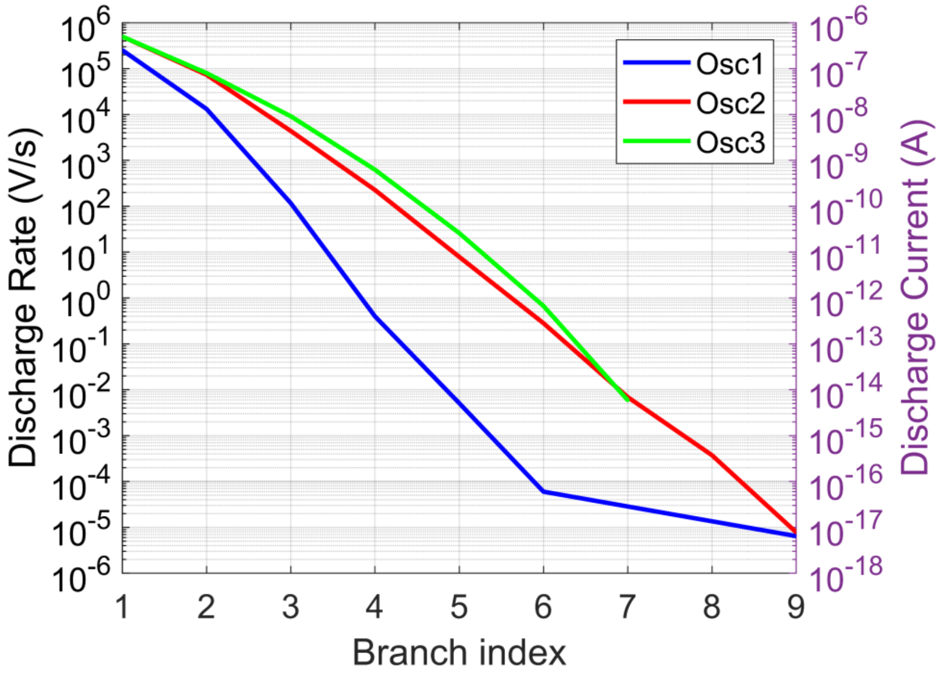

Supplementary Fig. 6. Measured discharge rate of three sawtooth oscillators (left axis) and inferred discharge current (right axis).

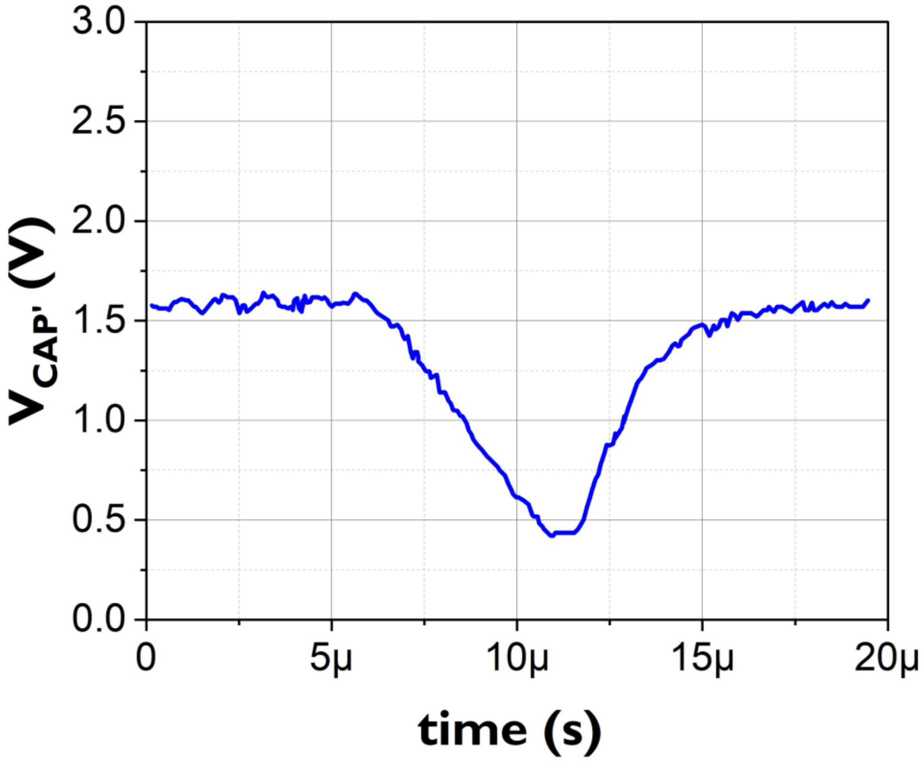

Supplementary Fig. 7. Osc1 branch <1> discharge rate of 252.5kV/s.

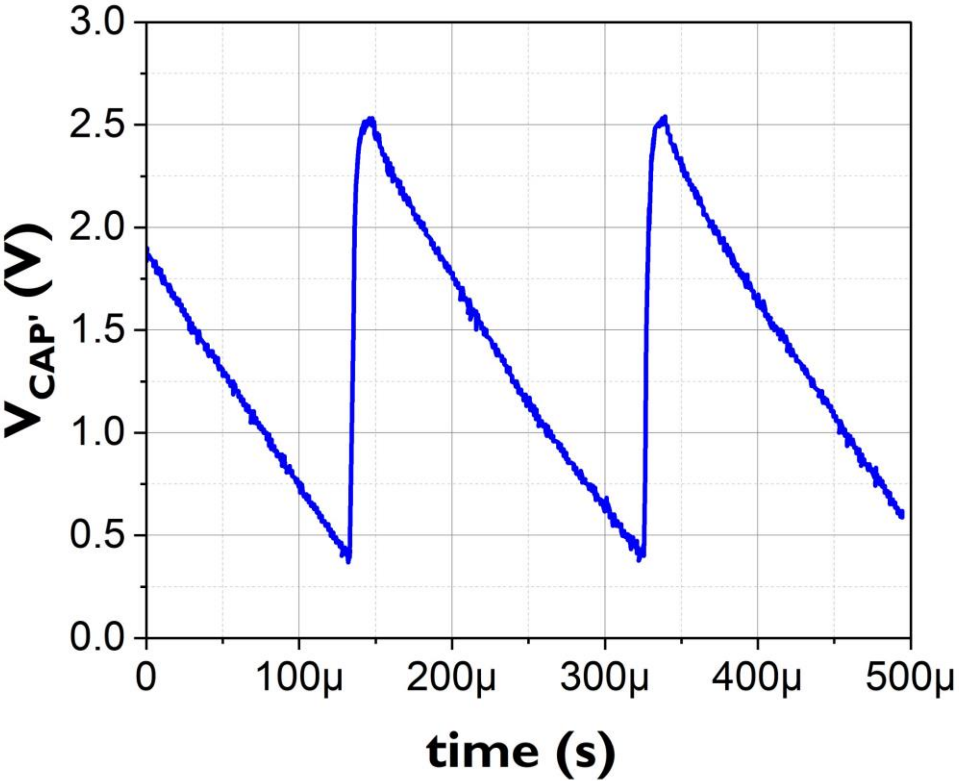

Supplementary Fig. 8. Osc1 branch <2> discharge rate of 13.2kV/s.

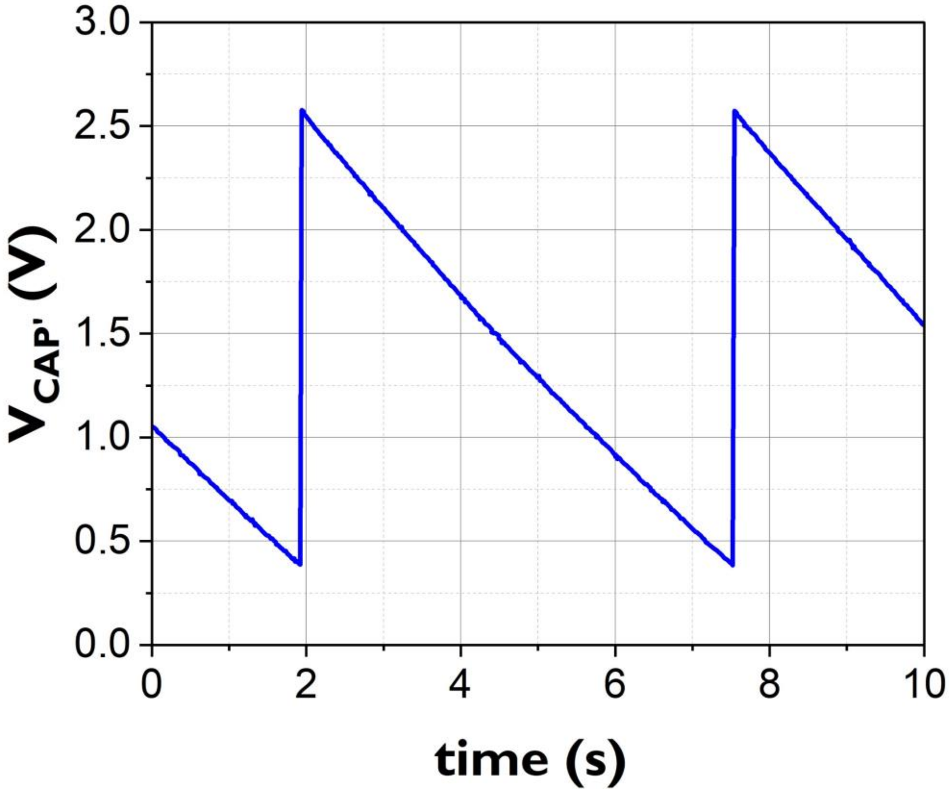

Supplementary Fig. 9. Osc1 branch <4> discharge rate of 397mV/s.

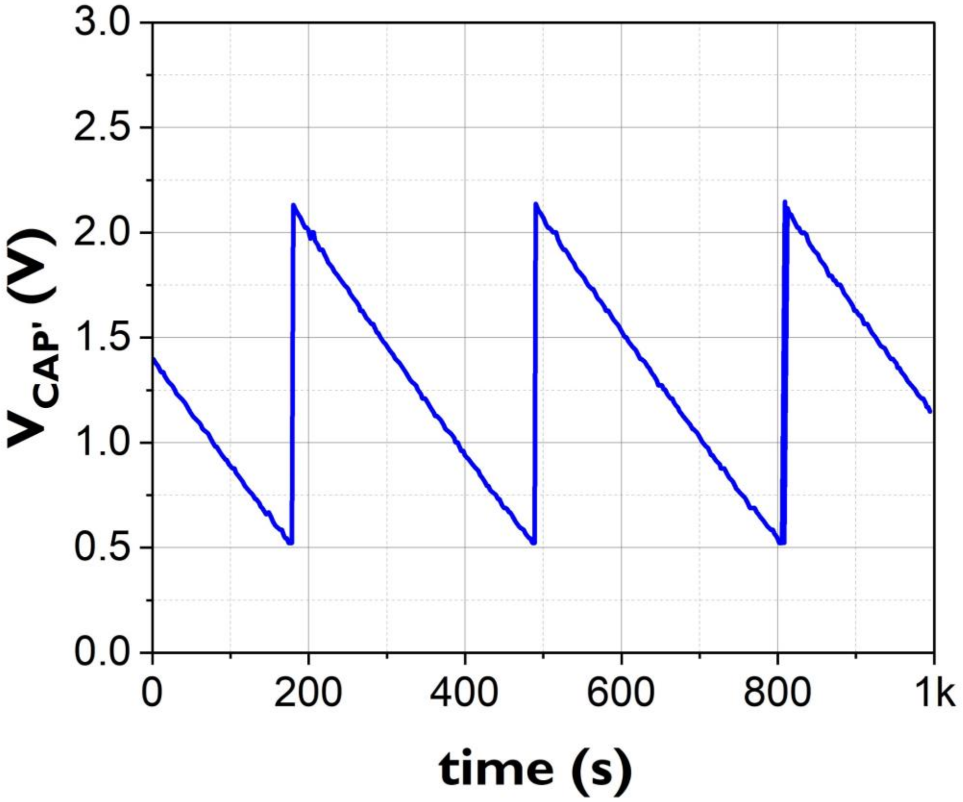

Supplementary Fig. 10. Osc1 branch <6> discharge rate of 5.24mV/s.

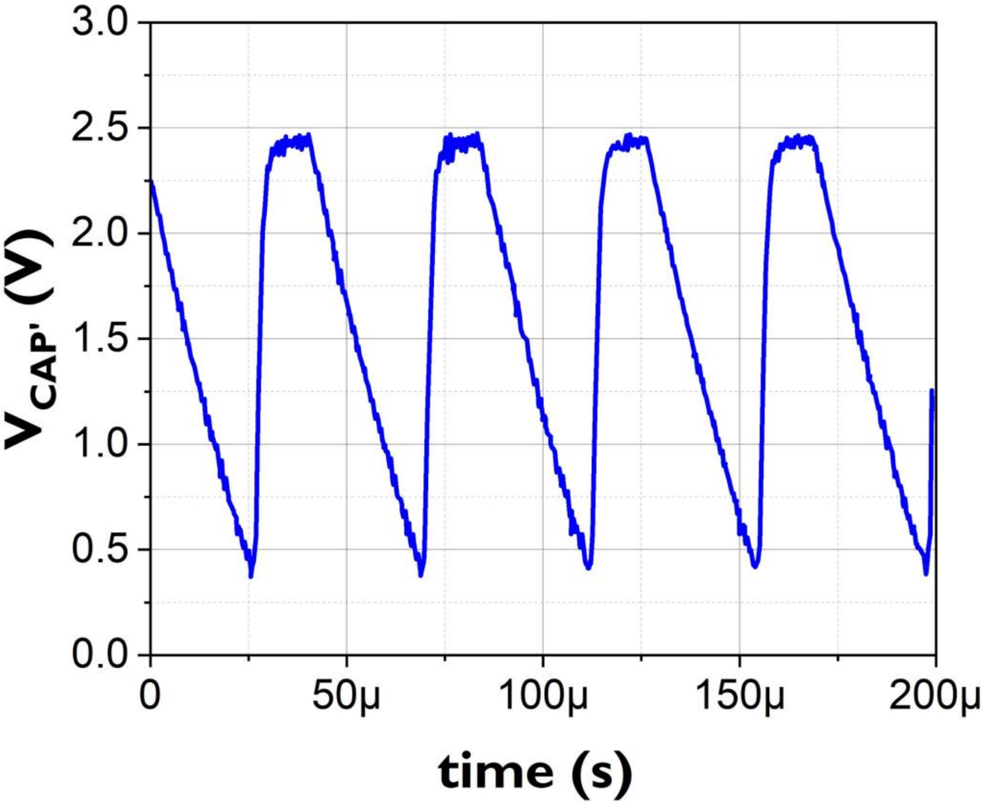

Supplementary Fig. 11. Osc2 branch <2> discharge rate of 73.5kV/s.

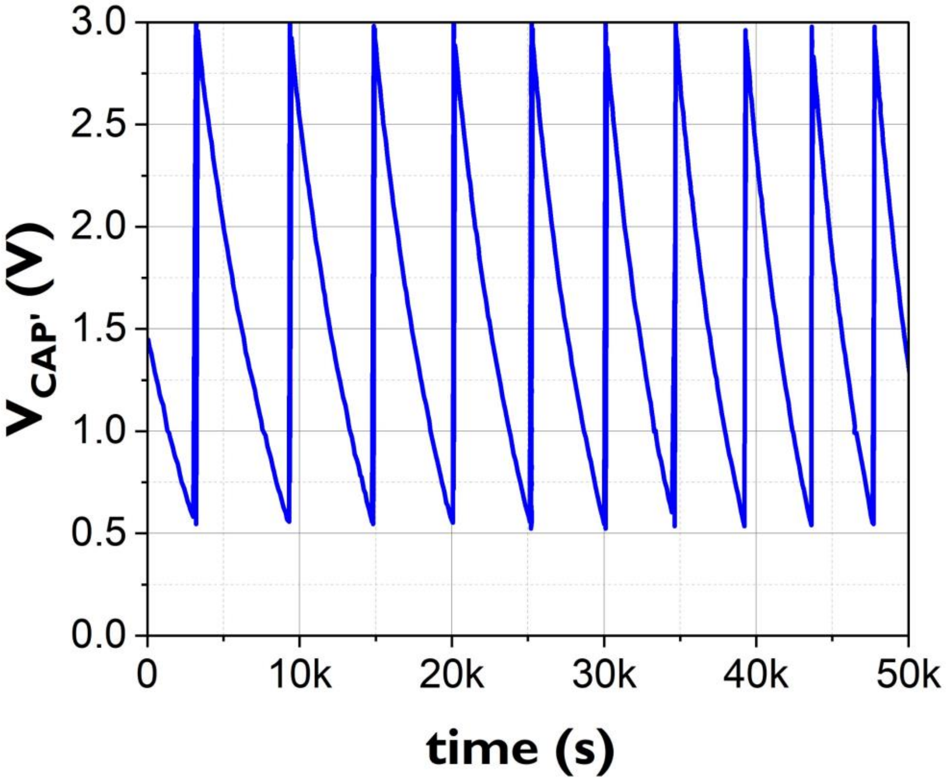

Supplementary Fig. 12. Osc2 branch <8> discharge rate of 374uV/s (at the end of the tails). Total measurement time is 50ks (13 hours 53 minutes).

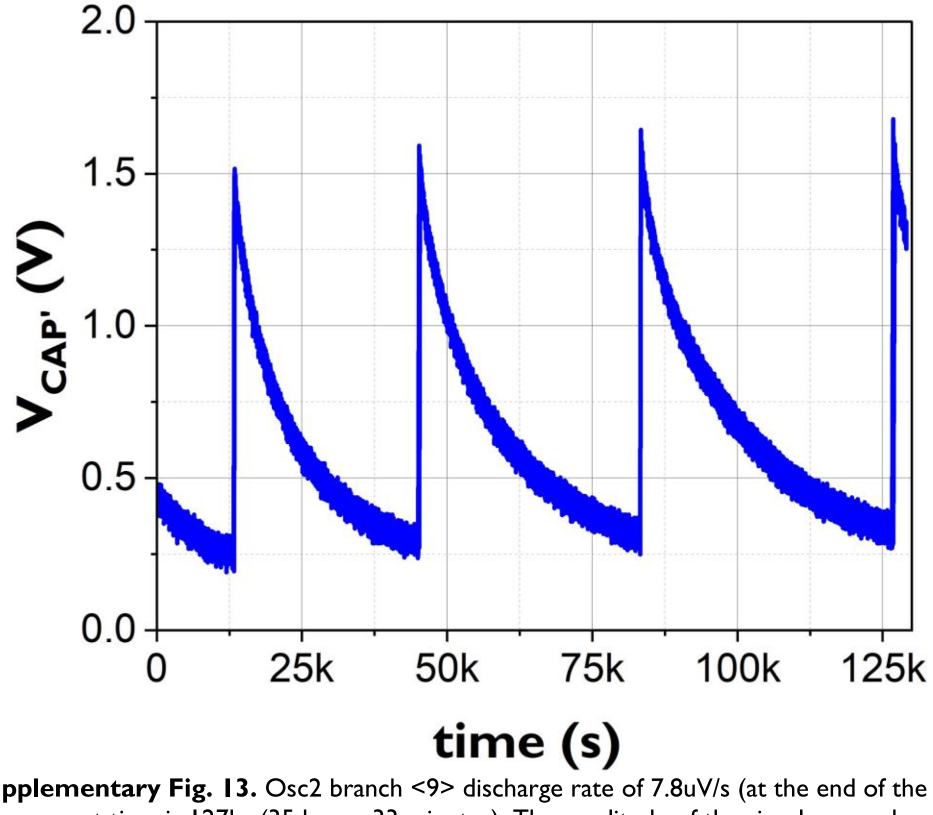

Supplementary Fig. 13. Osc2 branch <9> discharge rate of 7.8uV/s (at the end of the tails). Total measurement time is 127ks (35 hours 33 minutes). The amplitude of the signal was reduced to prevent degradation using the supply voltage V_{DD} and pin gnd_A.

Degradation of minimum leakage currents

As shown in the previous paragraphs, the characterization of the extremely low discharge currents, down to few atto amperes, required extremely long measurements. We observed that when trying to repeat such measurements with the same oscillator over several days, degradation effects were observed, resulting in a gradual increase of the minimum discharge currents. This is for example illustrated in Fig. 14 for Osc2 branch<9>. During a time-span of about 5 hours we captured an already degraded current of 792aA at the beginning of the capture until 2.98fA after 5 hours. Degradation continued during about a week until stabilizing at 120fA. We suspect this was probably due to a rather high supply voltage we were using of 6V. This was fixed during the neuron measurements which were made at 5 V which help preserve the samples.

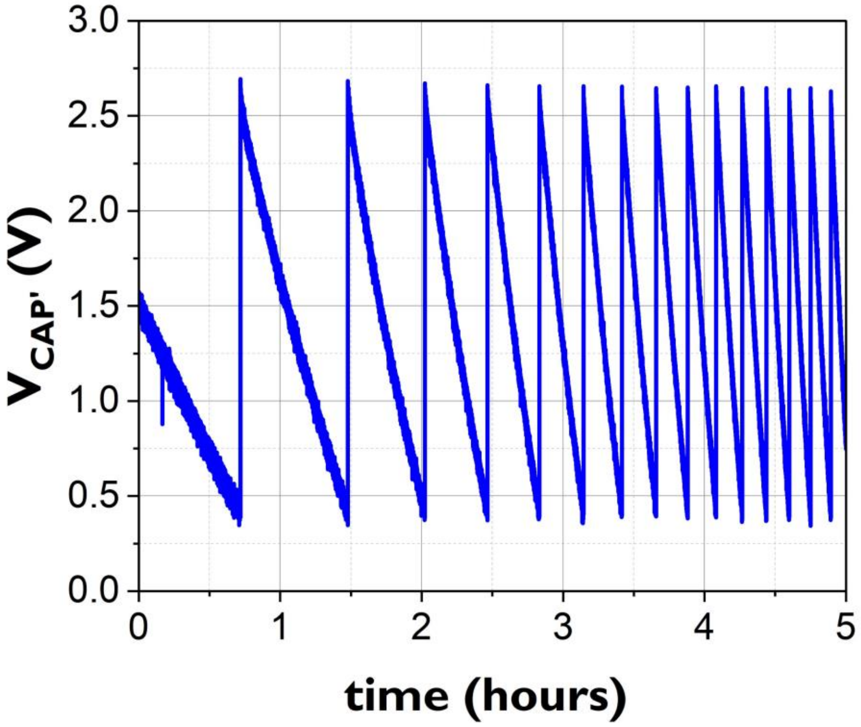

Supplementary Fig. 14. Osc2 branch <9> discharge current observed degradation after several days of operation. The amplitude of the signal was not reduced for this test.

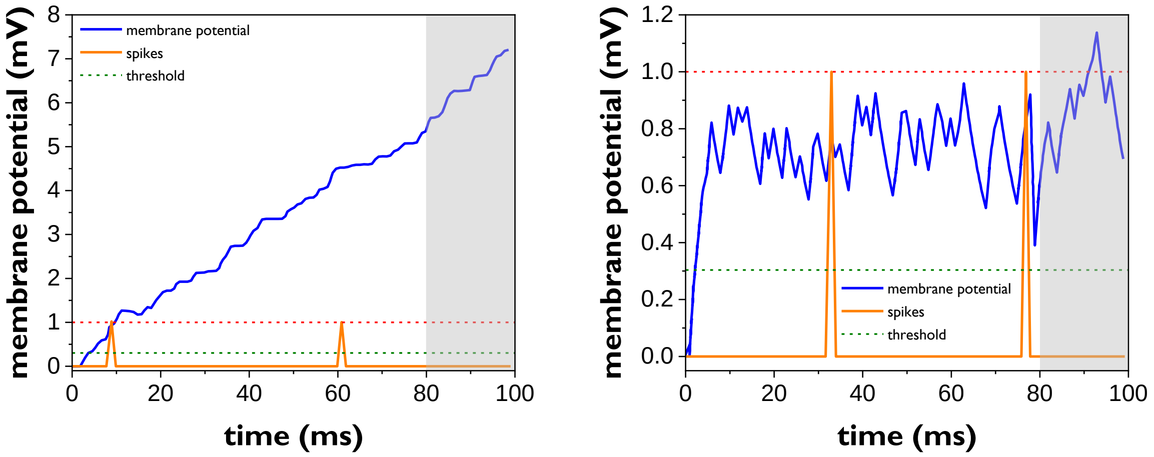

Supplementary Fig. 15. Membrane potential against time for a specific neuron in a recurrent SNN. **a.** Neuron with long memory time constant. **b.** Neuron with short memory time constant.

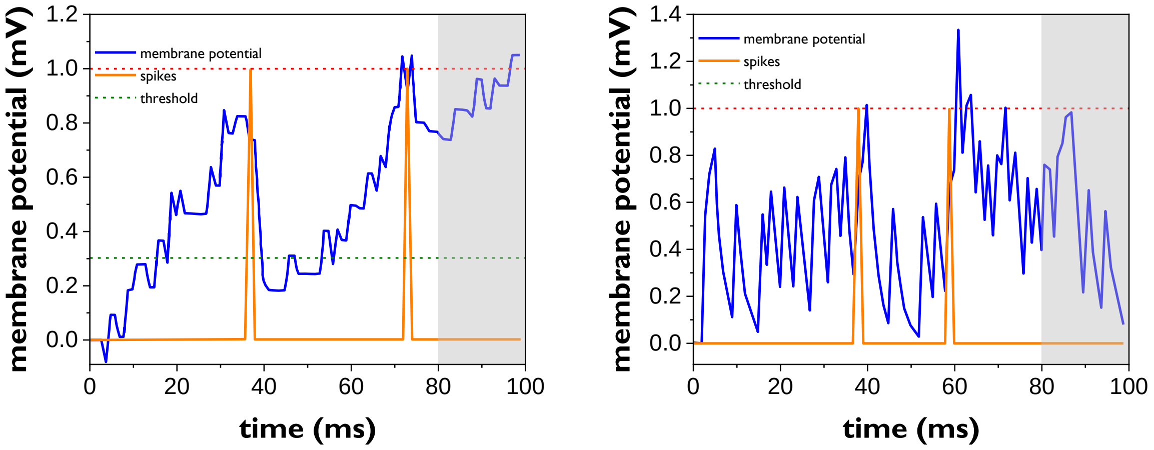

Supplementary Fig. 16. Membrane potential against time for a specific neuron in a feedforward SNN. **a.** Neuron with long memory time constant. **b.** Neuron with short memory time constant.

References of Supplementary Materials

[jssc03] Linares-Barranco, B. & Serrano-Gotarredona, T. On the design and characterization of femtoampere current-mode circuits. *IEEE J Solid-State Circuits* **38**, 1353–1363 (2003).
